# From Ionic Nanoparticle Organic Hybrids to Ionic Nanocomposites: Structure, Dynamics, and Properties: A Review

**DOI:** 10.3390/nano13010002

**Published:** 2022-12-20

**Authors:** Argyrios V. Karatrantos, Clement Mugemana, Lyazid Bouhala, Nigel Clarke, Martin Kröger

**Affiliations:** 1 Materials Research and Technology, Luxembourg Institute of Science and Technology, 5, Avenue des Hauts-Fourneaux, L-4362 Esch-sur-Alzette, Luxembourg; 2 Department of Physics & Astronomy, University of Sheffield, Hicks Buildingv Hounsfield Road, Sheffield S3 7RH, UK; 3 Polymer Physics, Department of Materials, ETH Zurich, Leopold-Ruzicka-Weg 4, CH-8093 Zurich, Switzerland

**Keywords:** ionic interaction, hybrids, interface, interphase, polymer dynamics, reinforcement, viscoelasticity, self-healing, nanocomposites

## Abstract

Ionic nanoparticle organic hybrids have been the focus of research for almost 20 years, however the substitution of ionic canopy by an ionic-entangled polymer matrix was implemented only recently, and can lead to the formulation of ionic nanocomposites. The functionalization of nanoparticle surface by covalently grafting a charged ligand (corona) interacting electrostatically with the oppositely charged canopy (polymer matrix) can promote the dispersion state and stability which are prerequisites for property “tuning”, polymer reinforcement, and fabrication of high-performance nanocomposites. Different types of nanoparticle, shape (spherical or anisotropic), loading, graft corona, polymer matrix type, charge density, molecular weight, can influence the nanoparticle dispersion state, and can alter the rheological, mechanical, electrical, self-healing, and shape-memory behavior of ionic nanocomposites. Such ionic nanocomposites can offer new properties and design possibilities in comparison to traditional polymer nanocomposites. However, to achieve a technological breakthrough by designing and developing such ionic nanomaterials, a synergy between experiments and simulation methods is necessary in order to obtain a fundamental understanding of the underlying physics and chemistry. Although there are a few coarse-grained simulation efforts to disclose the underlying physics, atomistic models and simulations that could shed light on the interphase, effect of polymer and nanoparticle chemistry on behavior, are completely absent.

## 1. Introduction

**Figure 1:**
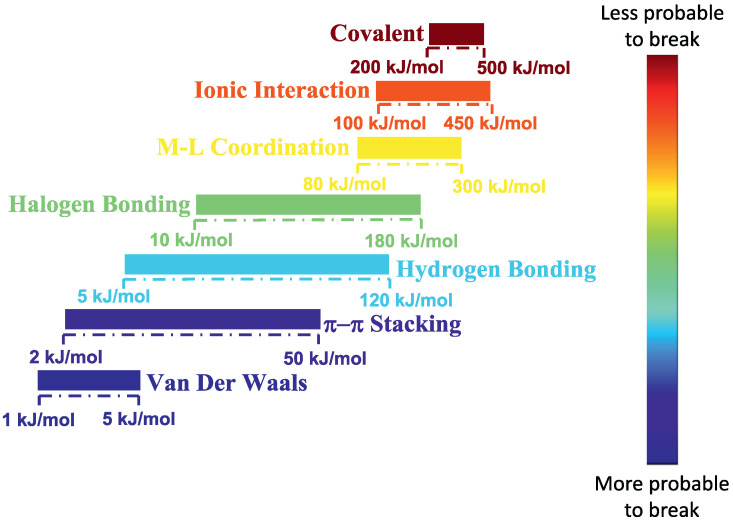
Energy ranges of different types of interactions, ranging from weak van der Waals to strong covalent bonds. Reprinted with the permission from Vereroudakis and Vlassopoulos [18].

Polymer nanocomposites that can contain dispersed either spherical, cylindrical, or plate-like nanofillers in a polymer matrix, have received increasing attention from industry and academia due to their improved macroscopic properties [1,2,3,4]. In nanocomposites, the interaction between the polymer and the nanoparticle surface is very important for processability of the nanocomposite and the material properties. A high degree of nanoparticle distribution and dispersion is necessary and is a prerequisite for an effective reinforcement in a polymer matrix. When polymer–nanoparticle interactions are relatively weak, nanoparticle aggregation is usually observed. Increasingly attractive polymer–nanoparticle interactions [5,6,7,8,9,10,11] (for instance from π–π interactions [12,13] to hydrogen bonding [6,14,15,16] and metal–ligand interactions [17], as can be seen in Figure 1), favor improved compatibility and promote dispersion.

A recent approach to dispersing nanoparticles into a polymer matrix is to let the interaction between nanoparticles and polymer chains be of an ionic (electrostatic) nature [19]. By using this approach the polymer–nanoparticle interactions are much stronger, and less likely to break than hydrogen bonding, etc. (Figure 1). Such an approach was first applied in ionically functionalized nanoparticles, such as nanosilicas, leading to solvent free dispersed nanofluids [20].

To the best of our knowledge, while there is a plethora of works on the topic of ionic nanoscale materials or organic hybrids covering almost 20 years of research, there is no review that comprises all the studies of such ionic nanomaterials focusing on the structure, dynamics and properties, except the reviews by Fernandes et al. [20], by Wang et al. [21], and Bhattacharya et al. [22] on synthetic strategies and applications of solvent-free nanofluids. Furthermore, nanocomposite behavior is strongly affected not only by polymer–nanoparticle interactions but also by nanoparticle characteristics (diameter, curvature, aspect ratio, surface shape, and functionalization), as well as nanoparticle loading.

Thus, the goal of this review is to discuss studies focusing on the structure and dynamics of nanomaterials that incorporate ionic (electrostatic) interactions between polymers and nanoparticles, and how this interaction can lead to improved material properties, such as mechanical, rheological, and self-healing properties. However, in this review we do not include studies where ionic liquids (ILs) have been blended with polymer nanoparticle mixtures [23,24,25,26,27] in order to improve the dispersion of the nanoparticles in the matrix. Nor do we consider nanocomposite materials in which ionic liquids have been used as compatibilizers [28,29,30] or plasticizers [31,32] nor nanofluids synthesized by polymer physical adsorption or covalent bonding, in order to achieve nanoparticle dispersion in the matrix.

In particular, we first review experimental studies on the structure, dynamics and properties of such ionic nanomaterials and, subsequently, we discuss simulation efforts to address the behavior of such nanomaterials. The paper is organized as follows: Section 2.1 discusses the structure and dynamics of such materials. In particular, we have split the discussion into systems that contain spherical (Section 2.1.1) or anisotropic (Section 2.1.2) nanofillers, using experimental techniques and approaches. Section 2.2 discusses the mechanical properties and performance of such ionic nanomaterials that can contain either spherical or anisotropic nanoparticles as nanofillers. Subsequently, we discuss the rheological and the self-healing behavior of the ionic nanomaterials in Section 2.3 and Section 2.4, respectively. Beyond the experimental works, in Section 3, we present simulation works that focus on addressing the structure and dynamics of ionic nanoparticle organic hybrids and ionic nanocomposites. Conclusions are offered in Section 4.

## 2. Experiments

### 2.1. Structure and Dynamics

In this section, we discuss only experimental studies that focus on the structure, dispersion, and dynamics of ionic nanoparticle organic hybrids (or nanoscale ionic materials) and ionic nanocomposites. In the first section, of spherical nanofillers, we first discuss experimental studies of systems that contain nanosilicas (SiO${}_{2}$) followed by studies containing carbon black, anatase (TiO${}_{2}$), zirconia (ZrO${}_{2}$) nanofillers, and, subsequently, systems containing very small diameter nanoparticles, such as polyhedral oligomeric silsesquioxane (POSS), fullerene, and metal nanoparticles, such as zinc oxide (ZnO), magnetic Fe${}_{3}$O${}_{4}$, gold (Au), palladium, etc. In the second section, we consider anisotropic nanofillers, such as multiwall carbon nanotubes (MWCNTs), gold (Au) nanorods or graphene. In Table 1, we tabulate all the different nanofillers and functional groups of the nanofiller or canopy (polymer) in ionic nanomaterials.

#### 2.1.1. Spherical Nanofillers

**Figure 2:**
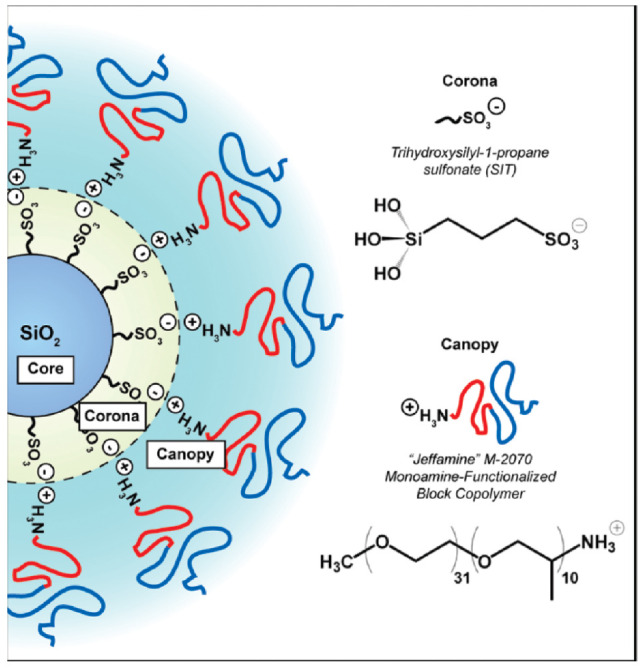
Schematic of the general structure for nanoscale ionic materials (NIMs) used in this study. The covalently attached corona has terminal sulfonic acid functionality. The canopy consists of a low molecular weight, amine-terminated diblock copolymer. Reprinted with permission from Jespersen et al. [35].

The synthesis of ionic nanofluids involves two steps: (i) the functionalization of the nanoparticle surface with a charged corona and (ii) the grafting of an opposite charged canopy by an ion-exchange reaction. One of the first works on nanoscale ionic materials (NIMs) by Bourlinos et al. [33] studied ionically modified nanosilicas with large counter anions (sulfonate, isostearate) at two volume fractions (13% and 27%) forming a viscous liquid and a glass, respectively. However, they cannot become crystalline solid materials. The glass transition temperature ($T_{g}$) and, hence, the local dynamics of these systems are governed by the large counter anions, whereas the flow properties are governed by the spatial correlation between the nanosilica particles (by tuning the volume nanosilica cores fraction and local interactions between polymer segments in the soft corona). X-ray scattering revealed a liquid-like ordering of the cores which significantly influenced their macroscopic flow properties [33]. Such ionic hybrid-based silica nanoparticles with a corona (possessing terminal sulfonic acid functionality) covalently grafted to the nanosilica [34] core and interacting ionically with an amine-terminated ethylene oxide/propylene oxide block copolymer canopy [35] is depicted in Figure 2.

**Figure 3:**
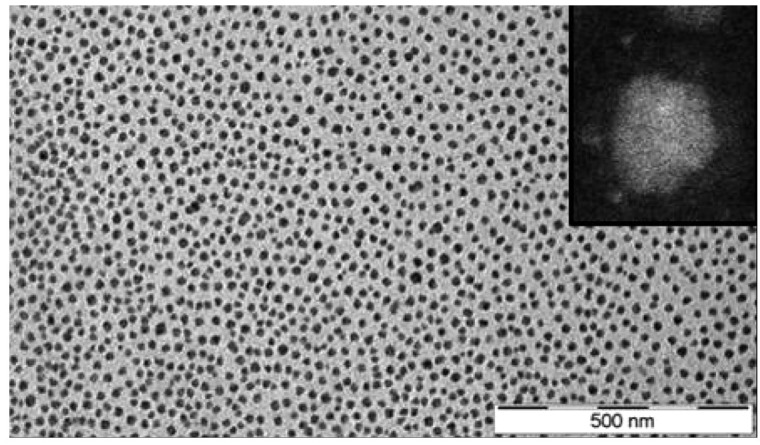
TEM images of the nanoscale ionic material (with inset showing a high-angle annular dark-field (HAADF) image of a single particle with a polymeric shell). Reprinted with permission from Fernandes et al. [36].

In a similar fashion, ionic nanosilica particle (with the anion charge carried by the surface hydroxyl) and amine-terminated poly(ethylene oxide) (PEO) (as a cationic canopy) showed a very high degree of dispersion state of the nanosilica in the PEO matrix as was evidenced by small angle X-ray scattering (SAXS) and transmission electron microscopy (TEM) experiments (such as the TEM image depicted in Figure 3).

In another study of functionalized nanosilica with a charged corona and ionically tethered oligomer canopy, the ionic nanofluid presented excellent friction-reducing and antiwear properties, when blended with polythylene glycol (PEG) [37]. Moreover, novel nanosilica ionic liquids were prepared by grafting nanosilicas with corona with terminal ammonium functionality, such as 3-(trimethoxysilyl) propyl ammonium [38]) that were interacting anionically with a long chain canopy layer (sulfonate-terminated PEG covalently connected to a nonylphenyl tail) [38] or with sulfonated alkyl chains [39,40]. Such NIM showed a liquid-like behavior at 10–55 °C exhibited spherulites formed at the microscale [38]. In a different case, 3-Glycidyoxypropyl-trimethoxy-silane grafted on the nanosilica core and the epoxy group reacted to tri-n-butylamine to form hydroxyl intermediate which was ionically tethered to sulfonated PEG canopy [41]. TEM images showed a core-shell structure (nanosilica cores were separated by a shell) and monodispersed quasi-spherical nanosilicas of diameter 9–11 nm [41]. The high reactivity of the hydroxyl group of the grafted chains on the ionic nanosilicas could react with −N=C=O groups in polyurethane prepolymer leading to crosslinking (the ionic nanosilica material acted as supramolecular crosslinking agent) with a polyurethane matrix forming an inorganic/organic hybrid membrane [41]. Another effort of ionic acrylate-modified (reactive) silica nanofluid with tetraacrylate monomer (tetrahydroxyethyl pentaerythritol tetraacrylate (THPETA)) was also implemented [42]. TEM image analysis exhibited low polydispersity of core nanosilicas of 9.7 nm diameter, revealing a more uniform dispersion of the ionic nanofluid than the acrylate-modified nanosilicas [42]. Furthermore, nanosilica cores grafted with 3-(trihydroxysilyl)–1-propane-sulfonic acid (SIT) and ionically tethered to polyoxyethylene octadecylamine (Ethomeen18/25) were formulated [43]. The content of nanosilica was up to 25% showing monodispersity upon heating [43]. In addition, a porous liquid based on a hollow nanosilica (of 14 nm inner diameter) functionalized with organosilane moeity (corona), whose molecular size was about 2 nm, and tethered by a negative sulfonated poly(ethylene glycol) (PEG) canopy was synthesized and fabricated [44]. The hollow nanosilica spheres had a mesoporous shell that could block species larger than 1.9 nm [21,44]. In addition, other hollow nanosilica particles, of different diameter, grafted with SIT corona ionically tethered to a Jeffamine (M-2070) canopy were prepared [45].

Increased chain ordering and weaker intermolecular interactions in the nanoparticle organic hybrids, compared to unbound polymers, were observed and were made more pronounced by lowering the grafting density of the canopies [46]. This distinct configuration of the grafted polymer chains on the nanoparticle caused a different carbon dioxide (CO${}_{2}$) packing and CO${}_{2}$ induced swelling behavior as was observed by Petit et al. [46]. Different types of canopy materials (either linear or branched) were used to functionalize the ionically functionalized nanosilica enabling the tuning of the CO${}_{2}$ sorption [47,48,49,50]. These can be synthesized either by having an ionic nanosilica core (grafted with a corona: either 3-(glycidyloxypropyl) trimethoxysilane or 3-(trihydroxysilyl)-1-propane sulfonic acid) with monoamine-terminated polytheramine as the canopy or having no grafted corona (the surface of nanosilica was protonated by using HCR-W2 ion-exchange resin) and using polyethylenimine or tertiary amine polyether as the canopy [47]. The structure–property relationships were investigated by focusing both on the contribution of the canopy and the core on the thermal stability, swelling behavior, and CO${}_{2}$ absorption properties of such NIMs [51]. It was concluded that the ionic bond between the canopy and the corona, and the covalent bonds between the corona and the nanoparticle surface significantly improved thermal stability compared to the polymer–nanoparticle mixtures. Moreover, a smaller canopy length and a larger core fraction could further enhance the thermal stability of the ionic nanomaterial [51]. Such ionic nanomaterials swelled less when heated or when they adsorbed CO${}_{2}$ compared to the polymers [51].

In another recent work, the impact of the bond type and grafting density on the thermal, structural, and transport behavior of nanoparticle organic hybrid materials was investigated [52]. Jeffamine (M-2070) canopies were tethered to SiO${}_{2}$ nanocores via ionic bonding. It was shown by small-angle neutron scattering (SANS) that tethered, interacting, or free polymers were present in aqueous solutions of such nanoparticle organic hybrids [52,53,54]. A large portion of free polymers appeared in the solution and were not bound to the functionalized nanosilicas [53]. The addition of electrolytes to the solutions altered the conformation of grafted and free polymers. In particular, the electrolytes decreased the size of the free polymers and the grafted polymer layer thickness [53] . The cation of the electrolyte competed with the amine of the tethered polymer (canopy), replacing the tethered polymer and, thus, causing the detachment of the canopy from the nanoparticle surface [53]. For instance, zinc (Zn${}^{+2}$) has a low binding energy with the ether oxygen of Jeffamine, which allows the movement of “free” Zn${}^{+2}$ from the bulk to the surface [54]. Moreover, nanosilica modified with coronas were implemented to decorate cotton fibers using electrostatic interaction. The resultant composites of silica and cellulose were characterized by field emission scanning electron microscopy (FESEM) and TEM revealing that layers of the nanosilica core-corona were deposited on cotton fibers and were more uniform than those of colloidal nanosilica particles. Such an improvement was mostly due to the enhanced dispersion and stabilization capabilities of the covalently grafted corona on the nanosilica particles. The adsorption amount of nanosilica modified by a corona was strongly controlled by the charge density of the nanoparticle surface and electrostatic strength of the cellulosic substrates [55]. Solvent-free ionic nanosilica particles were uniformly dispersed in an epoxy matrix. The addition of even the low loading of such ionic nanofillers greatly improved the tribological performance of the epoxy [56,57]. Furthermore, TEM images disclosed a good dispersion of surface-modified sulfonated nanosilicas, of up to 20% wt loading, in entangled imidazolium functionalized polyurethane (im-PU) matrices [58] and up to 5% wt loading, in an entangled rigid imidazolium terminated (im-PLA) and soft poly[ϵ-caprolactone-co-D,Llactide] (im-P[CL-co-LA]) [59]. SAXS experiments were completed to measure the level of dispersion of sulfonated SO${}_{2}$ [60] that showed no sign of nanoparticle aggregation in the form factor [60]. In addition, scanning TEM (STEM) revealed that the sulfonated SO${}_{2}$ were well-dispersed, due to the ionic interactions, and the dispersion was not affected under mechanical loading, [60] in agreement with the SAXS measurements [60].

**Figure 4:**
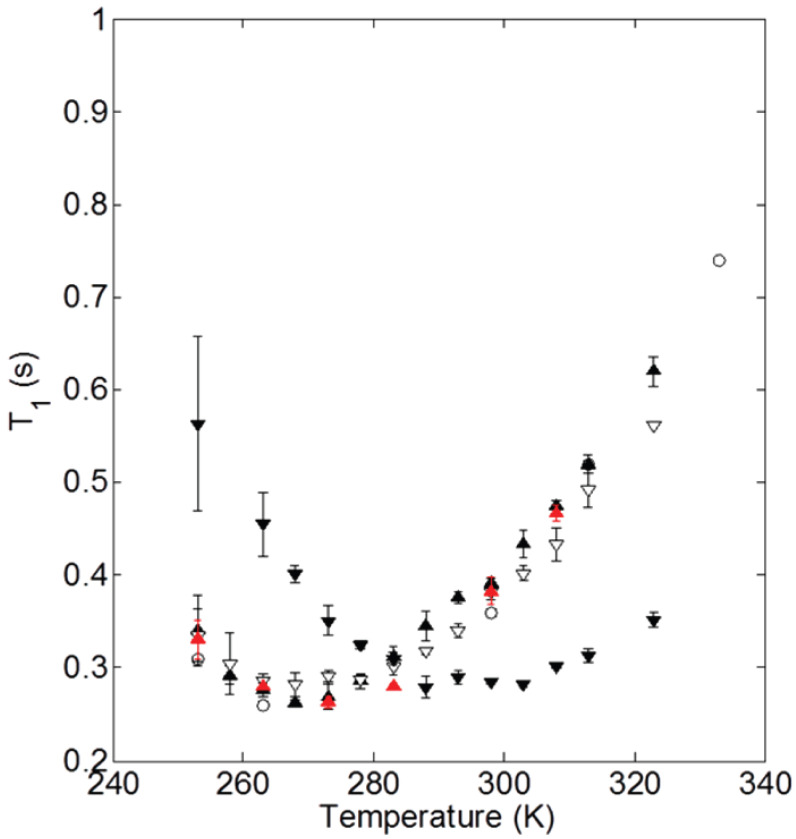
NMR spin-lattice relaxation times for M-2070 and M-600-based SiO${}_{2}$ NIMs as a function of temperature. The data are shown for M-2070 (∘) and SiO${}_{2}$ NIMs with 100% (filled ▴), 60% (open ▿), and 20% (filled ▾) neutralization of the SIT corona by M-2070 canopy. In addition, data are shown for 100% NIMs with one sodium cation per sulfonate group (red ▴). Reprinted with permission from Jespersen et al. [62].

Beyond the abundance of the structural behavior studies of ionic nanosilica organic hybrids, there have been a few efforts to study the dynamical behavior of the ionic cores and canopies in hybrid nanomaterials systematically. In particular, nuclear magnetic resonance (NMR) relaxation and pulse-field gradient (PFG) diffusion experiments were used to measure the ionic canopy (Jeffamine M-2070) dynamics, tethered to ionically modified (with trihydroxysilypropylsulfonic acid) silicas (18 nm diameter) [35,61,62]. It was shown that the block copolymer canopy was mobile and the fast dynamics (of the order of ns) was insensitive to the presence of the silica nanoparticles. The relaxation time $T_{1}$ shows a minimum at 263 K, not only for bulk Jeffamine (canopy) but also in cases of ionic nanomaterials with 100% and 60% neutralization of the corona with the canopy (Figure 4), showing that the chain dynamics are not affected by the presence of nanosilica cores. The minimum of $T_{1}$ for the case of 20% neutralization of the corona by the canopy shifted from 0.26 to 0.28 s, denoted a more restricted chain dynamics for that particular ionic nanomaterial [62]. In addition, canopy diffusion in the NIM is slowed relative to the bulk canopy and is faster than the diffusion of the ionic cores. Such characteristics lead to a lower viscosity and flow properties of the NIM [61].

This shows that liquid-like behavior in NIMs is due to the rapid exchange of the block copolymer canopy between the ionically-modified silica nanoparticles. Although the first studies of ionic liquid-like hybrids were referred to as NIMs (synthesized via ionic acid-base reaction between amine and sulfonate groups of polymers and nanoparticles), a broader definition was established later as nanoparticle organic hybrids [47]. Further, pulse-field gradient nuclear magnetic resonance (PFG-NMR) spectroscopies were implemented to investigate the canopy dynamics of the ionic nanosilicas synthesized (of 7, 12, and 22 nm diameter) of different core diversity (single, ternary). The diffusion coefficient of such ionic nanosilica particles decreased and thermal diffusion increased with the quantity of the canopy content [63]. The ternary mixture of all three diameters nanosilica cores showed the largest diffusion coefficient due to the less steric constraints (space filling) because of the different core sizes [63]. Recently, a nanoparticle organic hybrid material of nanosilica core ionically bonded to a poly(ethylenimine) canopy was characterized by broadband dielectric spectroscopy (BDS) and nuclear magnetic resonance (NMR) spectroscopy to study its dynamics [64]. In particular, carbon-NMR relaxation showed that the fast (ns) dynamics was not influenced by the presence of the nanosilica cores. The local motion was reduced for the methylene groups located within the polyetherimine canopy, whereas the relaxation of methylene located on the chain end of the canopy showed good agreement with the β -relaxation process as identified in the BDS measurements. Following, the Stokes–Einstein formula, the diffusion of the ionic nanosilica cores was estimated, however its value was far below the experimental limits that could be measured by PFG-NMR [64]. The relaxation occurring at high temperatures had a non-Arrhenius activation energy and could be described by the Volger–Fulcher–Tammann (VFT) equation. In addition, α-relaxation, associated with the “interior” carbons of the canopy, had a much stronger temperature dependence than the β-relaxation [64]. When poly(ethylenimine) [65,66] was used as the canopy, interacting via ionic bonding with the nanosilica core, the CO${}_{2}$ capture kinetics increased dramatically [67]. The addition of 0.1 M salt (sodium chloride: NaCl) lowered the viscosity the nanoparticle organic hybrid by up to 90%, and decreased the hydrodynamic radius of the ionic nanosilica, thus increasing the self-diffusion coefficient of the ionic nanosilica [66]. This hybrid material showed a liquid-like behavior unlike “conventional” polymer nanocomposites (which do not contain ionic interactions) but exhibited a higher viscosity due to additional contributions from tethered polymer chains [66]. Another transport property, in particular the ionic conductivity of the composite polymer electrolytes (Jeffamine terminated with amino groups) dramatically increased when sulfonated nanosilica particles were added, resulting in a broader electrochemical stability window and higher lithium transference number [68]. Flexible poly(ethylene glycol) methacrylate (PEGMA) and poly(ethylene glycol) diacrylate (PEGDA) crosslinked copolymer electrolytes, synthesized on reversible addition-fragmentation chain-transfer (RAFT) polymerization, which interacted through with ionic functionalized nanosilicas via electrostatics, have also been prepared [69]. The inclusion of ionically functionalized nanoparticles enhanced polymer mobility and, thus, the ionic conductivity of the nanocomposite [69].

Mixtures of ionic nanoparticle organic hybrid materials with secondary fluids (such as water, toluene, chloroform, acetonitrile, and ethyl acetate) were prepared to investigate the effect of the secondary fluid on the diffusion and viscosity. The molecular ratio of the secondary fluid to the ionically tethered polymer canopy (Jeffamine M-2070) altered the effect of the secondary fluid on transport properties. The structure and conformation of the canopy was also dependent on the secondary fluid [70]. Furthermore, ionic nanocomposites were synthesized in one pot by dispersing ionic nanosilica particles charge-balanced by both Li${}^{+}$ ions and mono-amino-terminated polyether (PEO-b-PPO-NH${}_{3}^{+}$) in an oligomeric PEO matrix. The Li${}^{+}$ and PEO-b-PPO-NH${}_{3}^{+}$ ions led to enhanced Li${}^{+}$ transference number and compatibility with the PEO matrix [71]. That system was tuned from a liquid to gel-like as the nanosilica weight fraction percentage increased from 0 to 40%, while the conductivity remained almost constant [71]. Thermal analysis and flammability measurements indicated that the nanocomposite electrolytes decreased the rate of weight loss and heat release rate, and exhibited open-flame ignition resistance in the test conditions used in the study [71].

A carbon black derivative, covalently grafted by a charged polysiloxane quaternary ammonium and ion exchanged with a sulfonated PEG canopy, behaved as liquid-like at room temperature in the absence of a solvent [72]. TEM images revealed that the carbon black derivate appeared dispersed in monodisperse core–shell nanoparticles, in contrast to the agglomeration behavior of the original carbon black. The low viscosity of the carbon black derivative can provide the opportunity to produce ionic carbon black polymer composites with enhanced processability and homogeneous distribution of the particles [72]. The functionalization of anatase nanoparticles (TiO${}_{2}$) surface with a quaternary ammonium organosilane led to ionically-modified nanoparticles that combined through electrostatic interaction with a polyethylene glycol (PEG) sulfonated anion (as the canopy) and exhibited liquid-like behavior, in solvent free [73] TiO${}_{2}$ nanoparticles with surface hydroxyl groups, treated by trimethoxysilane, which was synthesized by the sol-gel method [74]. The nanofluid was a yellow liquid of low viscosity at room temperature in the absence of solvent. By coating the TiO${}_{2}$ nanoparticles with an organic canopy, a better dispersion was achieved [74]. Different organosilane coronas (such as DMAC, TSAC and ILs) were used to functionalize TiO${}_{2}$ nanoparticles and interacting ionically with the sulfonated canopy PEG [75]. All the nanoparticle structures were core shell and dispersed well [75]. Moreover, zirconia nanoparticles (ZrO${}_{2}$) were functionalized with organophosphorus coupling agents bearing permanently charged functional groups (either cationic quaternary ammonium or anionic sulfonates) forming nanoparticles with a diameter below 10 nm. Such nanoparticles with stable surface charges could be used as building blocks for layer-by-layer-based techniques by using the electrostatic interaction [76] and were used to decorate the surface of larger, negatively charged (sulfonated) silica (SiO${}_{2}$) submicroparticles [76].

Liquid-like ionic POSS organic hybrids were also synthesized by octaammonium POSS nanoparticles ionically tethered to sulfonated or carboxylic polymer chains showing very good thermal stability [77]. Fully protonated fullerenes neutralized with amine terminated polyethylene/polypropylene oxide oligomers (Jeffamines) (fullerol ionic liquid) [78]. Such fullerol ionic fluids did not require a charged molecular corona attached to the fullerene core. The ionic bonding perturbed the thermal transitions and crystallization behavior of the amine [78].

**Figure 5:**
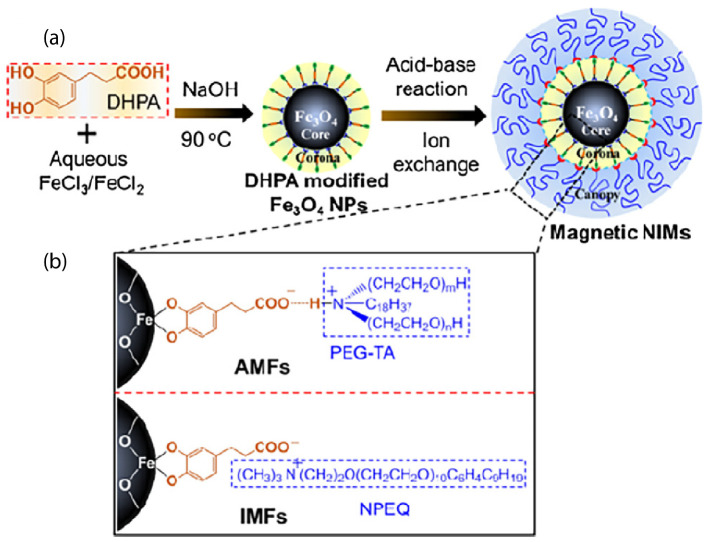
(**a**) Schematic showing the fabrication of Fe${}_{3}$O${}_{4}$ hybrids, (**b**) the triple structures of the two types of hybrids of Fe${}_{3}$O${}_{4}$ nanoparticle cores linked with PEG-TA canopy (denoted as AFMs) and NPEQ canopy (denoted as IMFs), respectively, by DHPA corona. Reprinted with permission from Li et al. [81].

The reaction of a positively charged organosilane with surface hydroxyl groups on the ZnO nanoparticles led to a permanent covalent attachment to the surface. A counterion was present to balance the charge and form a waxy solid or a hybrid organic–inorganic fluid, in the absence of a solvent, with a high quantum yield photoluminescence [79]. Furthermore, maghemite (γ-Fe${}_{2}$O${}_{3}$) nanoparticles were functionalized by a cationic organosilane (4 nm diameter) that interacted ionically with a sulfonate anion and yielded a liquid at room temperature [39,40]. Magnetic measurements confirmed the magnetic nature of such an ionic nanomaterial, denoting the first example of a ferrofluid [39]. Further studies focused on solvent-free magnetic Fe${}_{3}$O${}_{4}$ magnetic nanoparticles (of 2–3 nm diameter) whose structure behavior could be controlled by changing the corona structure [80]. Three kinds of surface corona modifiers were chosen which had the same functional groups but different lengths and quantities of alkyl chains [80]. The same sulfonated PEG counterion was used as the canopy. In another case, magnetic Fe${}_{3}$O${}_{4}$ nanoscale ionic materials, using a mussel-inspired bifunctional ligand of 3,4-dihydroxybenzenepropanoic acid (DHPA), were synthesized as can be seen in Figure 5, using a simplified one-step aqueous co-precipitation method [81]. Such a synthetic strategy is simple and can be extended to prepare various solvent-free nanoscale ionic materials. The microstructure, thermal stability, and phase transfer behavior can be easily tuned [81].

Two kinds of ionic nanofluids (AMFs and IMFs), depicted in the TEM images. In the case of AMF nanofluids, there was an ionic hydrogen bonding interaction. Both of these nanofluid types presented core-shell structures, as indicated by the distinct boundary between the inorganic crystallized core and the organic amorphous shell. The coated organic shell ensured the dispersion of the nanoparticles [81]. Other metal nanoparticles, such as platinum, gold, palladium, and rhodium, were functionalized with a thiol-containing ionic liquid. Such metal nanofluids exhibited liquid-like behavior at room temperature [82]. In particular, solvent-free gold nanofluids [83] were synthesized using a two-step process. This method comprised the grafting of the carboxylate-terminated thiol, 11-mercaptoundecanoic acid (MUA), onto the surface of gold nanoparticle via chemisorption and was followed by electrostatic self-assembly with a PEG-substituted tertiary amine [84]. Such functionalization can provide the material with conductivity [84]. Other efforts of ionic gold or lead nanoparticles functionalized with mercaptoethanesulfonate ionic liquid as the corona were reported for ionic nanofluids with improved electrical performance and durability [83]. Molybdenum disulphide (MoS${}_{2}$) nanoparticles were functionalized with 3-(trihydroxysilyl)-1-propane sulfonic acid (SIT) by condensation of hydroxysilyl groups of SIT and hydroxyl groups on the MoS${}_{2}$ surface and ionically tethered with oligomeric tertiary amine, as the canopy [85]. In a different study, the surface modification of MoS${}_{2}$ nanoparticles using a tetraethylene glycol-based ionic liquid (containing a chelating moiety attached to the cation and bis (trifluoromethane)sulfonimide (TFSI) as the anion) [86] led to very stable dispersions or microemulsions [86]. In a contribution by Gu et al. [87], MoS${}_{2}$ nanoparticles covalently grafted with organic corona (3-(trihydroxysilyl)-1-propane sulfonic acid: SIT) and tethered with ionic oligomeric PEG tertiary amine as the canopy, through an ion-exchange reaction, also showed a uniform dispersion of the metallic cores [87,88]. MoS${}_{2}$ ionic solvent-free nanomaterials exhibited Newtonian flow behavior, indicating their potential application for the lubrication of micro- and nano-electromechanical systems (MEMs/NEMs) [85]. The MoS${}_{2}$ nanofluids in the form of thin films could protect the substrates from scratching and wear [87].

**Figure 6:**
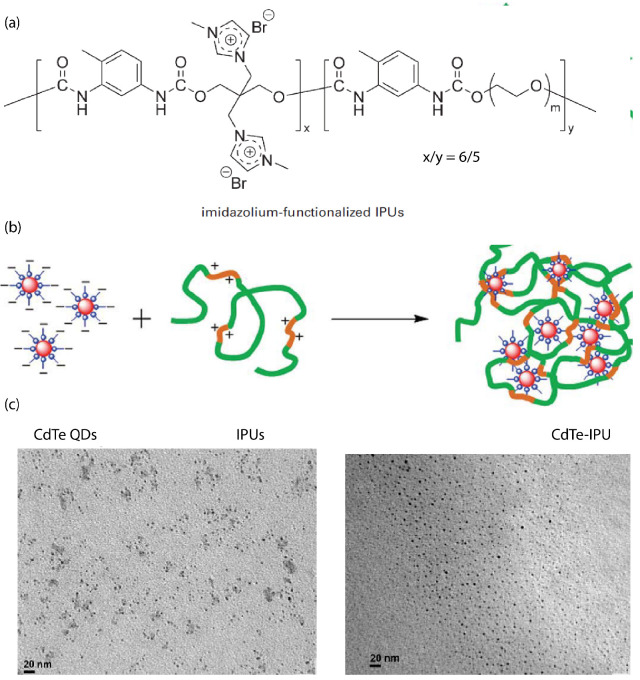
Quantum dot ionic polyurethane (iPU)—nanocomposite; (**a**) Imidazolium functionalized ionic Polyurethanes (iPUs). Reprinted with permission from Li et al. [89] (**b**) schematic preparation process of the quantum dot ionic polyurethane (iPU)—nanocomposite, **(c**) TEM images of the nanocomposite. Reprinted with permission from Li et al. [89].

The synthesis of imidazolium-functionalized ionic polyurethane (iPUs) (depicted in Figure 6a) combined with CdTe quantum dot (QDs) nanocrystals (3 nm of diameter) formed ionic nanocomposites through the electrostatic interaction between the positively charged iPUs and the negatively charged aqueous QDs [89]. TEM images showed that the QDs were uniformly monodispersed (Figure 6). The photochemical stability and strong fluorescent emission of CdTe–oPU nanocomposites was evidenced by both ultraviolet-visible (UV-Vis) absorption and photoluminescence spectra [89]. Solvent-free active carbon quantum dot (CQDs) grafted with poly(sodium 4-styrene sulfonate) (PSS), interacting with a Jeffamine (M-2070) as canopy, were also prepared [90].

Ionic nanomaterials based on CdSe/CdS/ ZnS core/shell QDs, anionically (carboxylate) functionalized, through a simple, rapid extraction method, and ionically interacting with long-chain cationic surfactant nonylphenyl poly(ethylene glycol) quaternary ammonium (NPEQ) were synthesized, facilitating the dispersion of solvent-free fluxible QDs (F-QDs). Such F-Qdots presented an efficient luminescence [91]. Lead-salts (PbS-QDs) were functionalized by using ionic liquids with thiol moieties as capping ligands [92]. These QDs were amphiphilic liquids exhibiting a fluid-like behavior, even at room temperature, and their photostability was dramatically improved compared to the synthesized oleic acid-capped QDs dispersed in toluene [92].

In the following section, concerning anisotropic nanofillers, we first refer to studies of systems that contain multi-wall carbon nanotube (MWCNT) nanoparticles, followed by systems that contain graphene types of nanoparticles and, subsequently, other types of nanofillers.

#### 2.1.2. Anisotropic Nanofillers

**Figure 7:**
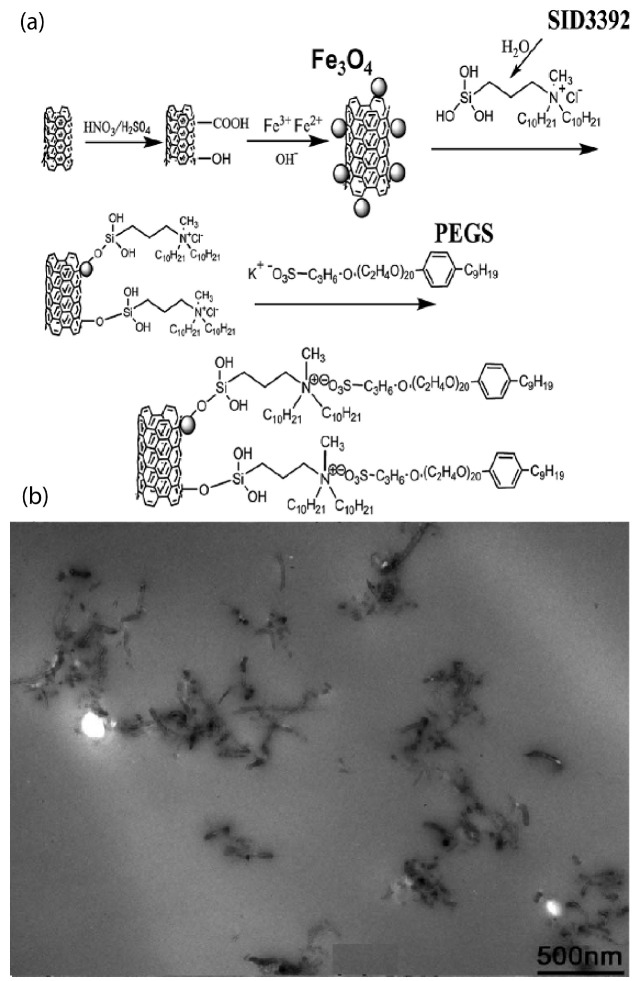
(**a**) MWCNT derivative, (**b**) TEM image of the liquid-like MWCNT derivative/epoxy nanocomposite. Reprinted with permission from Zheng et al. [97].

Although most studies on ionically functionalized nanoparticles focus on spherical type of nanoparticles, such as nanosilica, anatase, zirconia, POSS, fullerenes, there has also been a substantial synthetic effort focusing on functionalization, structure, dynamics, and properties, with anisotropic types of nanofillers, such as MWCNTs and graphenes. Such research was not discussed in the previous review by Fernandes et al. [20] despite its interesting behavior and properties. In particular, ionically functionalized long MWCNTs (100–500 nm long) were synthesized in a three step process [93]. These steps are: (i) functionalization with polar hydrophilic groups (OH, COOH, or C=O), (ii) reaction with a polysiloxane quaternary ammonium salt with the OH or COOH group, and (iii) reaction of the resulting molecules with sulfonate salts via an ion-exchange reaction to form the ionic MWCNT nanomaterial [21,93]. The resulting ionic MWCNT is different to the MWCNT derivative synthesized by a functional CNT–amine reaction method, with a PEG-substituted tertiary amine [94]. In both studies, ionic MWCNTs presented a liquid-like behavior at room temperature and were highly dispersible in both aqueous and organic media. Similar to work by Bourlinos et al. [94] solvent-free MWCNT nanofluids with ionically tethered poly(ether amine)-terminated polymers were successfully prepared and evaluated as sorbents for CO${}_{2}$ uptake [95]. It was found that the synergistic effect between MWCNTs and polyetheramine help to enhance the CO${}_{2}$ capture capacities of the sorbents compared to unbound polyetheramine and pristine [95]. In another work, nanoscale ionic, solvent-free, MWCNTs were covalently grafted with a charged polysiloxane quaternary ammonium salt and reacted with a PEG-functionalized sulfonate salt [96]. The liquid-like behavior of these MWCNT derivatives came from their relatively high amount of organic materials and the continual motion of the large organic ions [96]. Thermal properties, as well as the temperature-dependent and strain-dependent viscoelasticity of MWCNTs were related to the microscopic structure of their coating [96]. Moreover, a MWCNT derivative was synthesized by attaching Fe${}_{3}$O${}_{4}$ to the surface of MWCNT and employing tertiary amine terminated organosilanes as a corona and sulfonated PEG salt as a canopy [97], following a similar synthetic approach to that of Ref. [98]. Such a nanoscale ionic material exhibited liquid-like behavior at room temperature. TEM images verified the good dispersion of the MWCNT derivative (Figure 7) in the solvent and the epoxy matrix [97].

**Figure 8:**
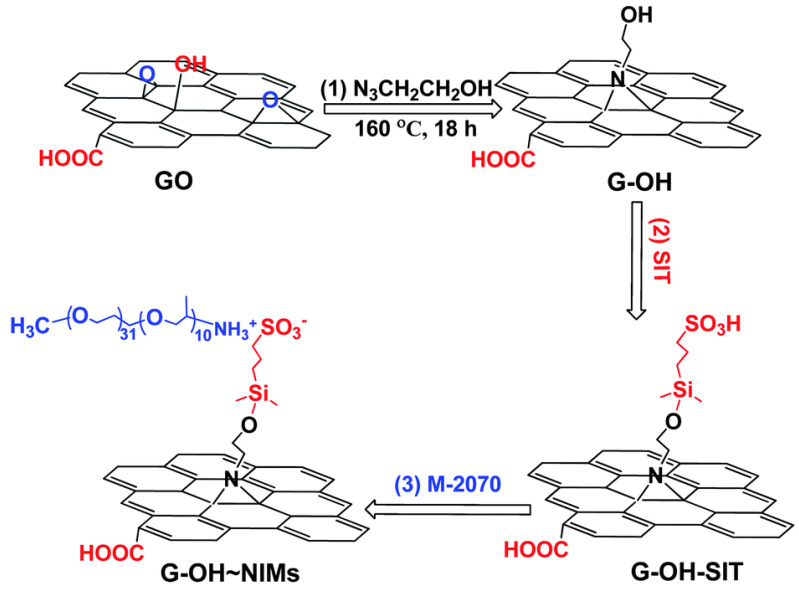
Schematic of the G-OH nanoscale ionic materials: (1) graphene oxide is surface-functionalized with 2-azidoethanol to obtain hydroxyl-rich graphene (G-OH); (2) G-OH is modified by condensation with a sulfonate organosilane (SIT) to prepare the acidic graphene (G-OH-SIT); and (3) G-OH-SIT is neutralized with the basic amino-terminal block copolymer M-2070 via acid–base reactions. Reprinted with permission from Wu et al. [104].

Furthermore, ionic-acid treated CNTs and ionically tethered with PEG tertiary amine were a viscous liquid at room temperature and were stable and homogenously dispersed in a polyurethane matrix, forming a nanocomposite material [99]. Another type of ionic functionalization on MWCNTs was used by Lan et al. [100] who prepared solvent-free ionic and well dispersed MWCNTs nanofluids within epoxy matrix. The conductivity of this nanoscale organic hybrid exhibited a percolation threshold of approximately 0.408 wt%. This small amount of nanofluid improved the mechanical properties of the epoxy nanocomposite [100]. The use of a MWCNT-based solvent-free nanofluid improved the dispersion of MWCNT in epoxy resin and adjusted the interface strength between MWCNT and the matrix. The investigation showed the effect of the polyetheramine on the properties of the epoxy [101]. Gold nanorods ionic nanocomposites were synthesized by step-wise surface modification having sulfonic acid as corona and ionically tethered to end-terminated with an amine group Jeffamine canopy [102]. This ionic nanofluid could flow at room temperature and was sensitive to external stimuli, such as mechanical shearing [102]. These specific corona and canopy were implemented on hydroxide perovskite (MnSn(OH)${}_{6}$) nanoparticles, forming a solvent-free ionic nanofluid with a thread-like morphology, showing good dispersity, fluidity at room temperature, and very good processability [103]. Such nanosale threads were dispersed in an epoxy matrix and curing agent (METHPA) forming a nanocomposite [103]. Graphene-based nanoscale ionic materials were synthesized from hydroxyl-functionalized graphene (G-OH) [104,105] as depicted in Figure 8. First, the G-OH graphene was modified by condensation with a sulfonate organosilane (SIT) to prepare the acidic graphene (G-OH-SIT). Then, it was neutralized with the cationic amino-terminal block copolymer (canopy: Jeffamine M-2070 polyetheramine) (Figure 8). Such G-OH-based nanoscale ionic materials showed an amphiphilic behavior, in that they can be dispersed and stabilized, for months, in both water and organic solvents [104,105].

**Figure 9:**
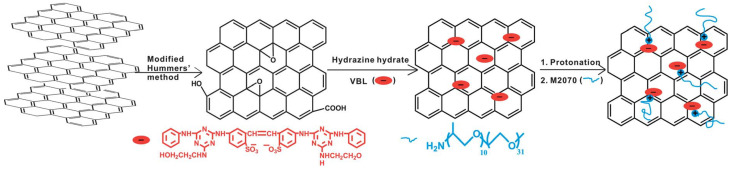
Schematic of the graphene NIM using a fluorescent whitening agent, VBL, as a non-covalent modifier and Jeffamine M-2070 as canopy. Reprinted with permission from Tang et al. [107].

Ionic graphene/nanosilica organic hybrids were synthesized by attaching nanosilica (SiO${}_{2}$ of diameter 100 nm), which was functionalized by SIT, on graphene oxide (GO), and ionically tethered to Jeffamine M-2070 [106]. Such an ionic organic hybrid showed very good dispersion stability, as characterized by TEM [57]. Another graphene ionic nanoparticle organic hybrid was synthesized, that could also be dispersed in a variety of solvents, by taking a different approach using a non-covalent modifier, through the chemical reduction of graphene oxide (GO) using a fluorescent whitening agent, VBL, and interacting with electrostatic interaction with bulky Jeffamine M-2070, as can be seen in Figure 9 [107]. In another study, the sulfonation of GOs was prepared with sodium sulfanilic acid and ionization with bulky amine-terminated Jeffamine (canopy) [108]. The supramolecular ionic liquid that was formed exhibited excellent solubility and amphiphilicity, confirming the liquid-like behavior by rheological measurements [108]. Such nanoscale ionic liquid material could be a promising route for the fabrication of GO composites by changing the canopy molecule [108]. Moreover, the introduction of multivalent cations can successfully enhance the interfacial strength of graphene based-nanocomposites by forming ionic crosslinking networks with GO nanosheets by coordination. Combined with other type of interfacial interactions, such as hydrogen bonding, π–π stacking, etc., the synergistic effect resulted in the improvement of nanocomposite performance, including that of thermal, electrical, fatigue-resistant, and mechanical properties [109].

A nanoscale liquid-like graphene/Fe${}_{3}$O${}_{4}$ hybrid was first prepared by using sulfuric acid-terminated organosilane (SIT) as the corona and monoamine-terminated polyetheramine (Jeffamine M-2070) as the canopy [98]. First, the Fe${}_{3}$O${}_{4}$ nanoparticles were deposited on the graphene sheet and, secondly, the organosilane was grafted onto the surface of the graphene/Fe${}_{3}$O${}_{4}$ hybrid to form the corona. The ionic fluid exhibited paramagnetic properties, and was behaving as a Newtonian fluid at room temperature, presenting excellent amphiphilicity and electronic conductivity [98]. Such ferrofluids could be used as a graphene lubricant and could be promising for the production of supercapacitors, batteries [110] and nanocomposites [98]. In addition, graphene/Fe${}_{3}$O${}_{4}$ nanoparticles were grafted with tertiary amine terminated organosilanes as a corona and sulfonated PEG salt as a canopy [110,111], following a similar synthetic approach to that of Ref. [98]. Such liquid-like nanocrystal, solvent-free, functionalized graphene was uniformly dispersed up to 1 wt% in an epoxy matrix [111].

Other types of ionic nanoparticles, such as calcium carbonate (CaCO${}_{3}$), were functionalized by grafting charged polysiloxane quaternary ammonium salt which then reacted with sulfonated PEG salt [112] through an ion-exchange reaction. TEM images showed that the ionic CaCO${}_{3}$ nanomaterial had a rhombohedral shape with a well-defined core-shell structure, of 20–50 nm size. The aggregation of this CaCO${}_{3}$ can be avoided by the functionalization. The shell ensures the stabilization of nanoparticle dispersion and enables the inorganic core to move smoothly. In a complementary effort of CaCO${}_{3}$ nanoparticle synthesis, via an in situ formation method, nanoparticles with a polysiloxane quaternary ammonium chloride (PQAC) corona were formed followed by an ionic exchange reaction to fabricate a poly(ethylene glycol) 4-nonylphenyl 3-sulfopropyl ether potassium (NPEP) canopy [113]. TEM images also confirmed the rhombohedral shape, in this case, with a well-defined core-shell structure, and also showed a NPEP canopy with a thickness of 4 to 6 nm. X-ray powder diffraction confirmed that the CaCO${}_{3}$ inner core had a calcite crystalline structure, whereas the NPEP canopy was amorphous. That canopy was found to show characteristic crystallization-melting behavior in the presence of the ion bonding, as characterized by the differential scanning calorimetry (DSC) technique. A thermogravimetric analyzer (TGA) indicated that the CaCO${}_{3}$ ionic nanoparticles achieved a high content of NPEP canopy, as well as an improvement in thermal stability due to the ion-bonding effect [113]. Such CaCO${}_{3}$ nanoparticles also presented a liquid-like behavior in the absence of solvent [113]. A similar synthetic approach, via tertiary amine (N,N-decyl-N-methyl-N trimethylsilyl propyl ammonium chloride: DMAC) and sulfonate PEG anion canopy (nonylphenoxy poly(ethyleneoxy) ethanol sodium sulfonate: NPES), was used to functionalize sepiolite (magnesium silicate of fibrous morphology) nanoparticles, which also presented liquid-like behavior at room temperature [114]. Halloysite-based solvent-free ionic nanofluids were prepared, and had a polysiloxane quaternary ammonium corona ionically tethered to sulfonated functionalized PEG [115]. This ionic nanofluid also exhibited a liquid-like behavior at room temperature but with different dispersion and stability in different solvents. The fluidity could be tuned by altering the graft density of the shell to the core and the shell–core, interaction which can be tuned by core preheating or by changing the surface functional moieties of the core by acid or alkaline etching [115]. In other types of materials, by controlling the electrostatic attraction between positively charged poly(ethylene imine) (PEI) and negatively charged sodium alginate (SA) dispersion, the charge density of SA can be tuned to enable the good dispersion of Ca${}_{2}$Nb${}_{3}$O${}_{10}$ perovskite nanosheets in SA [116]. MXene is modified using an organosilane (OS) with acidic moieties, then an alkaline oligomer is electrostatically grafted to form the resultant stable electron-balanced MXene nanoparticle organic hybrids. The prepared Ti${}_{3}$C${}_{2}$T${}_{x}$ MXene hybrids were rather stable with a long antioxidant properties [117]. Furthermore, such MXenes exhibit macroscopic flow behaviors at room temperature, improving processability. Combining a poly(butylene terephthalate) (PBT) ionomer with montmorillonite clays results in the exfoliation of the clays due to strong electrostatic interactions between the charged surfaces of the silicate clay particles and the SO${}_{3}^{-}$Na${}^{+}$ groups of the PBT ionomer [118].

**Table 1:** Ionic nanoparticle organic hybrids and ionic nanocomposites.

Nanofiller	Nanofiller’s	Canopy or	Canopy’s Functional	Ref.
	Functionalization	Polymer	Group	
Silica	Ammonium	Isostearate	Carboxylic	[33]
Silica	Sulfonic acid	PEG	Tertiary amine	[34]
Silica	Sulfonic acid	polyetheramine (Jeffamine), PEO	NH${}_{3}^{+}$	[35,36,48,50]
Silica	Ammonium	PEG	Sulfonate	[38]
Silica	Ammonium	Alkyl chains (C13-C15)	Sulfonate	[39,40]
Silica	Ammonium	PEG	Sulfonate	[41]
Silica	Sulfonic acid	Ethomeen	Tert-amine	[43]
Hollow silica	Ammonium	PEG	Sulfonate	[44]
Hollow silica	Sulfonic acid	Jeffamine	NH${}_{3}^{+}$	[45]
Hollow silica	Carboxylic acid	PEG	Tertiary amine	[99]
Silica	Sulfonic acid	Polyurethane	Imidazolium	[58]
Silica	Sulfonic acid	Poly(lactic acid)	Imidazolium	[59,119]
<Silica	Sulfonic acid	PDMS	Ammonium	[120]
Carbon black	Ammonium	PEG	Sulfonate	[72]
Anatase (TiO${}_{2}$)	Ammonium	PEG	Sulfonate	[73]
POSS	Ammonium	PEG-based polymers	Carboxylic/Sulfonic	[77]
Fullerene	Hydroxyl	Jeffamine	NH${}_{3}^{+}$	[78]
ZnO	Ammonium	PEG-based copolymer	Sulfonate	[79]
γ-Fe${}_{2}$O${}_{3}$	Ammonium	Alkyl chains (C13-C15)	Sulfonate	[39,40]
Fe${}_{3}$O${}_{4}$	Ammonium	PEG	Sulfonate	[80]
Fe${}_{3}$O${}_{4}$	DHPA	PEG	Ammonium	[81]
Gold (Au)	Sulfonate	Ammonium chloride (Adogen)	Quarter-ammonium	[83]
Gold (Au)	Carboxylic acid	PEG	Tertiary amine	[84]
Gold (Au)	Sulfonate	tris(2-ethylhexyl)/triisooctyl/triisopentyl/		
		tripentyl/trihexyl/trioctylamine	Quarter-amine	[121]
Au nanorods	Sulfonic acid	Jeffamine	NH${}_{3}^{+}$	[102]
MnSn(OH)${}_{6}$	Sulfonic acid	Jeffamine	NH${}_{3}^{+}$	[103]
MoS${}_{2}$	Sulfonic acid	Ethomeen/PEG/	Tertiary amine	[85,86,88]
Quantum dot (QD)	Thioglycolic acid	Jeffamine		[89]
Carbon QD	Sulfonate	Polyurethane	NH${}_{3}^{+}$	[90]
MWCNT	Ammonium	PEG	Sulfonate	[21,93,94,95]
MWCNT+Fe${}_{3}$O${}_{4}$	Ammonium	PEG	Sulfonate	[97]
Graphene+Fe${}_{3}$O${}_{4}$	Sulfonic acid	Jeffamine	NH${}_{3}^{+}$	[98]
Graphene	Quarter-Amine	PEG	Sulfonate	[122]
Graphene+Fe${}_{3}$O${}_{4}$	Ammonium	PEG	Sulfonate	[111]
Graphene	Carboxylic/Sulfonic acid	Jeffamine	NH${}_{3}^{+}$	[104]
Graphene	Sulfonic acid	Jeffamine	NH${}_{3}^{+}$	[105]
Graphene	Carboxylic acid	VBL	hydroxyl (OH)	[107]
Graphene+SiO${}_{2}$	Sulfonate	Jeffamine	NH${}_{3}^{+}$	[106]
Calcium carbonate	Ammonium	PEG	Sulfonate	[112,113]
Halloysite (Hal)	Ammonium	PEG	Sulfonate	[115]
Fe${}_{x}$O${}_{y}$	Ammonium	Polyacrylate copolymer	Sulfonate	[123]
Peroskite nanosheet	Sodium alginate (carboxylated)	Poly(ether-imine)	Ammonium	[116]
Ti${}_{3}$C${}_{2}$T${}_{x}$ MXene	Sulfonic acid	Jeffamine	Ammonium	[117]
Montmorillonite clay	Quaternary ammonium	Poly(butylene terephthalate)	Sulfonate	[118]

### 2.2. Mechanical Properties

#### 2.2.1. Spherical Nanofillers

**Figure 10:**
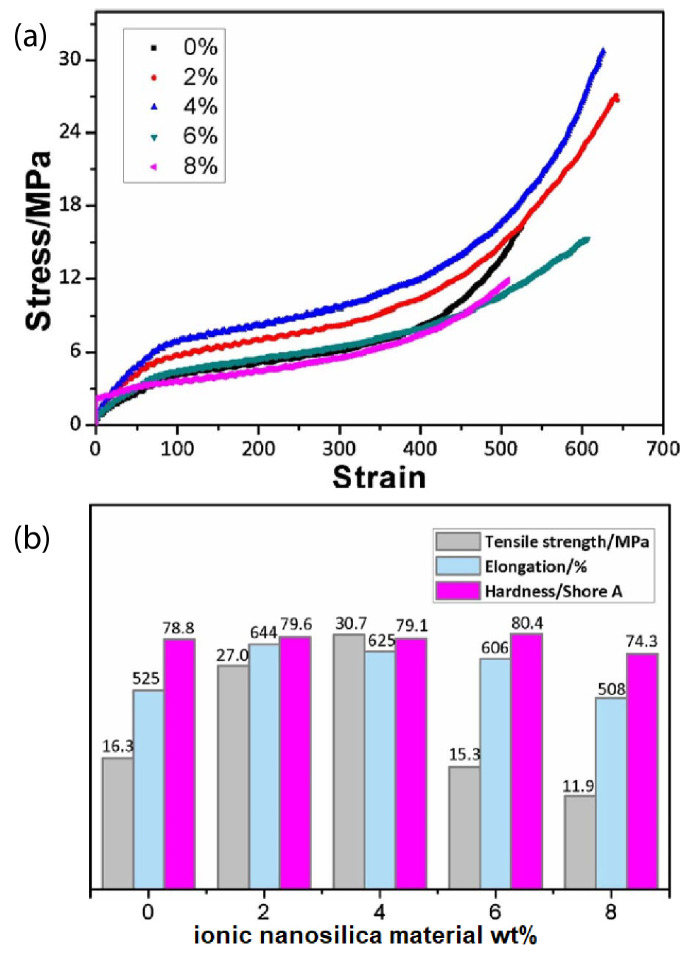
(**a**) Stress strain curves of PU/ ionic nanosilica hybrids, (**b**) Mechanical properties of PU/ ionic nanosilica hybrids. Reprinted with permission from He et al. [41].

Introducing ionic nanoparticles to the polymer matrix endows the nanocomposite obtained with excellent mechanical properties and performances. For example, the ionic interactions enhance the nanocomposite toughness, ductility, damping capacity, thermal stability, interfacial strength, strain at break, plasticity, shape recovery, and self-healing. For instance, the hybrid of ionically functionalized nanosilicas with reactive hydroxyls with polyurethane (PU) prepolymer showed improved mechanical properties, such as tensile strength, hardness, and elongation at break [41]. In particular, the tensile strength of the PU hybrid was 30.7 MPa (twice higher than that of PU matrix with was 16.3 MPa), the elongation was 625 % (larger than that in non-ionic PU nanocomposites [124,125]) by addition of 4 wt% ionic nanosilica and the hardness of PU hybrid reached a maximum of 80.4 °C when 6 wt% ionic nanosilicas were added as can be seen in Figure 10 [41].

For other reactive nanofluids, such as acrylate-modified nanosilicas, loaded at 1 wt%, to a THPETA resin can result in a decreased hardness modulus and increased storage modulus, and, thus, increase the toughnness of the nanocomposite [42,126].

**Figure 11:**
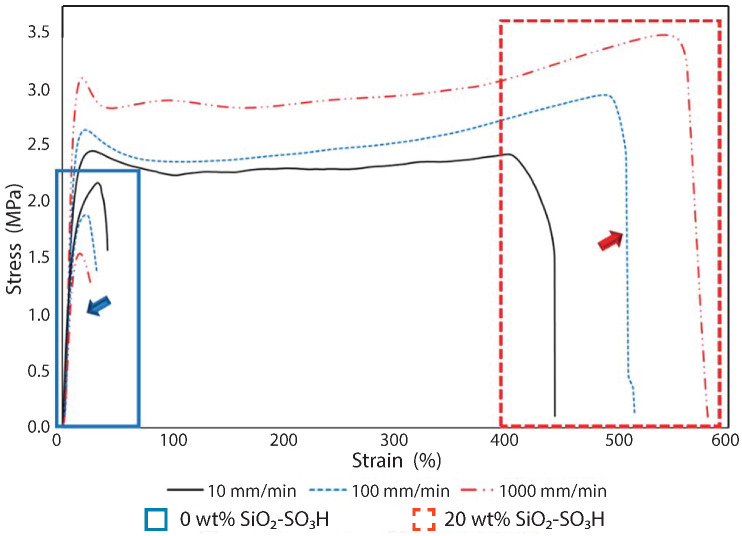
Stress–strain curves of polyurethanes (solid blue square) and the ionic PU nanocomposite containing 20 wt% of nanosilica (dash red square) at different strain rates: 10 mm/min (solid black line), 100 mm/min (dash blue line) and 1000 mm/min (dash dot red line). Reprinted with permission from Odent et al. [58].

**Figure 12:**
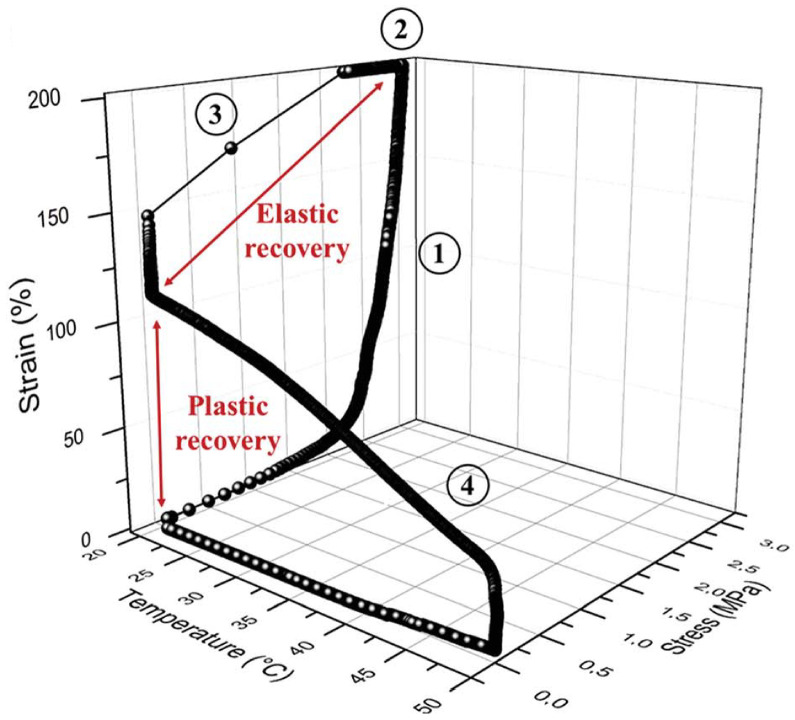
Cyclic stress-controlled thermomechanical test (DMTA) of the nanocomposite containing 20 wt% of nanosilica: (1) deformation, (2) fixation, (3) unloading and plastic recovery, and (4) heating and plastic recovery. Reprinted with permission from Odent et al. [58].

Recently, a new family of ionic nanocomposites by Odent et al. [58], Potaufeux et al. [60,119] based on ionically functionalized nanosilicas mixed with oppositely charged imidazolium (cationic)-functionalized polyurethanes (iPUs) presented impressive mechanical properties. These systems had an attractive electrostatic interaction [8,20,35,36,127,128,129] between nanosilicas and the iPU matrix. In particular, two main interesting features have been observed in iPU/nanosilicas composites: [58] (a) upon increasing the nanosilica mass fraction (up to 20 wt%) an increase in strain at break was observed; (b) by increasing the strain rate, a simultaneous increase in elastic modulus and toughness was detected [58,120]. While the increase in elastic (Young’s modulus) can be explained as being due to the addition of nanosilicas into the iPU matrix, the physical mechanisms that led to improvement of strain at break and toughness in nanocomposites remained still as open questions [130]. According to Odent et al. [58], imidazolium-functionalized polyurethane matrix reinforced by sulfonate-modified nanosilica improvements did not originate from the nature of the iPU matrix, but rather from the nanoparticles mobility during deformation, while ionic bonds break and reform with polymer chains as the nanoparticles move [131]. It was hypothesized that ionic nanocomposites dissipated strain energy through the dissociation of ionic crosslinks and nanoparticle motion [58]. The first mechanism was related to the ionic interactions [132] between imidazolium cations and sulfonate anions being dynamic, so breaking and reforming ionic crosslinks drove the stress–strain curve, as can be seen in Figure 11. It was proven that the reversible dynamic dissociation of the ionic crosslinks was responsible not only for most of these impressive performances of the nanocomposite but also for the recovery to its initial state after experiencing a large deformation (Figure 12) [58].

**Figure 13:**
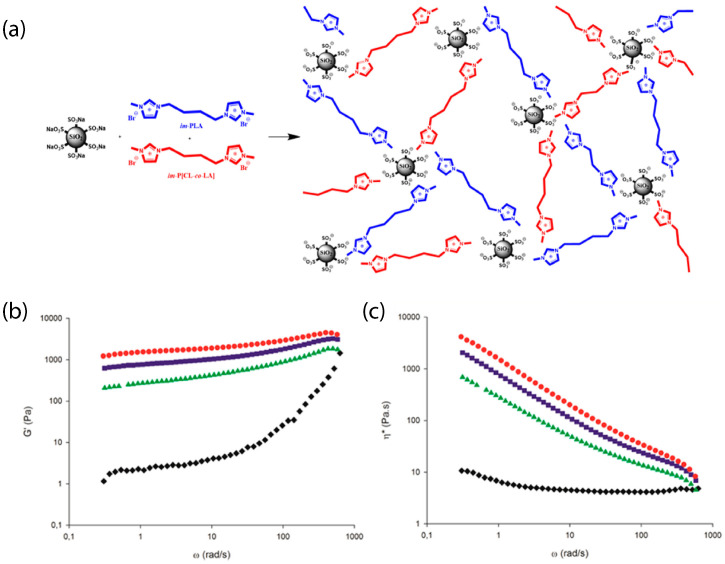
(**a**) Schematic of ionic organic hybrids based on PLA and imidazolium terminated PLA and P[CL-co-LA] oligomers and sulfonated nanosilicas. (**b**) Storage modulus $G^{\prime }$ and (**c**) complex viscosity $\eta^{*}$ as a function of the angular frequency at 180 °C PLA/im-PLA/im-P[CL-co-LA] (50/25/25 wt%) (black diamonds) and corresponding hybrids containing 1 wt% (green triangles), 3 wt% (blue squares), and 5 wt% (red circles) of sulfonated nanosilica. Reprinted with permission from Odent et al. [59].

Furthermore, mechanistic studies of ultra-tough polylactide nanosilica composites (Figure 13) showed on one hand, a unique property profile that combines ultra-toughness and ductility (up to 150%), without critical loss of stiffness and, on the other hand, improved thermal stability [up to 40 °C higher compared to neat poly(lactic acid) (PLA)] and shape-memory behavior [59,119]. In addition, the mechanistic study elucidated an energy-dissipative toughening mechanism in PLA/im-PU/SiO${}_{2}$-SO${}_{3}$H blends under quasi-static and high-speed loadings (ca.impact, tensile, 3-points bending) [119].

A recent study demonstrated the effect of ionic interactions in a poly(dimethyl-siloxane) (PDMS) matrix [120]. PDMS has an extremely low glass transition ($T_{g}$) and is utilized in a lot of applications due to its excellent thermal stability, high permeability, and good biocompatibility. As a liquid at room temperature, most applications require PDMS to be chemically crosslinked and/or combined with nanofillers to realize the requisite mechanical properties. The functionalization of the PDMS with ionic interactions (1.73 mmol/ g) and their cross-linking through oppositely charged silica nanoparticles (loading 20 wt%) had an impact on the glass transition which varied from −120 °C to approximately 80 °C. The effect of ionic interactions depended on the charge density and charged location as highlighted by the thermal analysis of ionic PDMS polymers, which evidenced a higher thermal stability for end-functionalized PDMS than cation-grafted PDMS. Similarly, the viscoelastic properties of these materials showed a clear evidence of a significant viscous response in the case of cation-grafted PDMS, as confirmed by frequency sweep experiments in a parallel plate rheometer, while end-functionalized PDMS exhibited solid-like behavior. A tensile testing analysis of the ionic nanocomposite specimen showed a better mechanical reinforcement with the highest break stress in the entangled PDMS (MW: 25 kg/mol $>M_{e}$) with a nanosilica loading of 10 wt%. The relatively low PDMS charge density allowed avoidance of the formation of kinetically trapped nanostructures. As the charge density of ionic PDMS increased inhomogeneous nanosilica distribution was observed [120].

Flexible poly(ethylene glycol) methacrylate (PEGMA) and poly(ethylene glycol) diacrylate (PEGDA) crosslinked copolymer electrolytes were synthesized by chain-transfer (RAFT) polymerization which endowed the polymer electrolyte membranes with flexibility and appeared an increased yield stress and tensile modulus when the ionically functionalized nanosilica were added to system [69]. Moreover, an experimental study guided by molecular simulation efforts was conducted by Ma et al. [133] to investigate the interfacial interaction between a water-borne polyurethane (WPU) and (negatively or positively) functionalized SiO${}_{2}$ microspheres. The hydrogen bond and electrostatic interaction mainly affected the elongation at the break of the nanocomposite, while the tensile strength was affected by the electrostatic interaction. The study also revealed that the surface characteristic of SiO${}_{2}$ was a key parameter to influence the interface interaction of the water-borne polyurethane (WPU) functionalized SiO${}_{2}$ nanocomposite.

#### 2.2.2. Anisotropic Nanofillers

The elastic modulus of ionic acid treated CNTs/PU nanocomposite decreased in comparison to the neat PU matrix, above the $T_{g}$, meaning that the rubbery plateau for the nanocomposite was lower than that of the PU matrix [99]. The breaking strength and the elastic modulus of ionic CNT nanofluid/PU nanocomposite were found to decrease slightly in comparison to the neat PU matrix, but, on the other hand, the elongation at break was increased by almost 100%, thus increasing the toughness, which can be attributed to the introduction of PEG oligomers that acted as plasticizers, decreasing the $T_{g}$ of the nanocomposite [99]. The processability and mechanical properties of polymeric matrices reinforced by CNT fluids have been studied by Li [134]. In particular, solvent-free ionic CNTs nanoparticles interacting with flexible counterions were incorporated into a polyamide (PA11) matrix to formulate a nanocomposite material. The soft organic coating and the flowability of the solvent free CNTs proved responsible for the performance and plasticization effect obtained. MWCNT-filled epoxy composites were also promising materials for structural damping materials. In particular, the interfacial strength and MWCNT dispersion significantly influenced the mechanical and damping properties [101]. Indeed, excellent dispersion and weak interface strength could simultaneously enhance the damping properties and mechanical properties of the epoxy. When 0.5 wt% of MWCNT was added to the epoxy matrix, the bending strength, bending modulus, and impact strength of the nanocomposites increased [101]. It has also been observed by a previous study [100] that the mechanical properties of ionic MWCNT nanofluids, such as bending modulus, strength, and impact toughness were simultaneously improved. In particular, when 0.5 wt% MWCNT was added to the epoxy matrix, the bending strength, bending modulus, and impact toughness were increased by more than 10%, 14%, and 40%, respectively [101]. In the absence of solvent, a functional liquid-like MWCNT derivative was dispersed in an epoxy matrix to formulate nanocomposites [97]. The MWCNT derivative could be aligned in the epoxy matrix with a magnetic field and the formed nanocomposite had an improvement on the impact toughness of pure epoxy by 194%, as well as an improved heat resistance in comparison to that of the epoxy [97]. The incorporation of ionic liquid-like nanocrystal-functionalized GO/Fe${}_{3}$O${}_{4}$ (at 1 wt%) into the epoxy matrix increased the impact toughness from 11.2 to 26.67 KJ/m${}^{2}$ (138.12% improvement in comparison to neat epoxy matrix) and enhanced the $T_{g}$ about 33 °C, due to the soft organic shell [111]. A similar increase in the impact toughness of flexible MnSn(OH)${}_{6}$ crystallite threads (at 1 wt%) in epoxy nanocomposites was also observed [103]. The core of MnSn(OH)${}_{6}$ crystallites played the role of reinforcing agent and the organic shell was considered as plasticizer [103].

### 2.3. Rheological Properties

#### 2.3.1. Spherical Nanofillers

In this section, we discuss studies focusing on the rheological behavior of ionic nanoparticle organic hybrids and ionic nanocomposites. In most ionic nanofluids, the incorporation of ionic charges leads to a liquid-like behavior at room temperature, but also has a strong effect on the rheological response of the nanomaterial.

In particular, ionic nanosilica organic hybrid (with the anion charge carried by the surface hydroxyl in the corona) and amine-terminated poly(ethylene oxide) (PEO) (as a cationic canopy) showed an improved flow behavior [36,41], behaving as a viscoelastic fluid in comparison to neutral PEO/nanosilica mixtures which yielded a solid [36]. Moreover, nanosilica ionic liquids grafted by a corona of terminal ammonium functionality that interacted anionically with a long chain canopy layer (sulfonate-terminated PEG covalently connected to a nonylphenyl tail) behaved as Newtonian fluid at high strain rates [38]. The loss modulus (G${}^{\prime \prime }$) surpassed storage modulus (G${}^{\prime }$) showing liquid behavior at room temperature [36,41]. Organic canopies moved freely and the viscosity of such ionic organic hybrids decreased with increasing temperature [36,41]. In addition, they were crystallized to spherulites (of 20–50 µm diameter) after a long time aging at ambient temperature, as was observed by a polarizing microscope (POM) [38]. This particular ionic nanofluid showed a complicated temperature dependent rheology in contrary to the anatase ionic nanofluid [73] which showed a viscosity decrease from 40 °C to 80 °C. In particular, at temperatures higher than 40 °C, the storage modulus $G^{\prime }$ exhibited a fast decay by more than three orders of magnitude. In addition, a remarkable increase appeared during cooling from 50 °C to 29 °C, exhibiting an hysteresis [38]. Such ionic nanofluid exhibited shear thinning at temperature ≤40 °C. [38] The addition of ionic nanosilica cores increased the viscosity of the nanoparticle organic hybrid relative to the polyetherimine canopy [45,64] and resulted in Newtonian behavior [64]. The mean relaxation times obtained from these measurements coincided with those of the structural relaxation for polyetherimine as determined from BDS measurements [64]. Moreover, a porous liquid based on hollow nanosilicas (of 14 nm inner diameter) functionalized with organosilane moiety, as corona, and tethered by a negative sulfonated poly(ethylene glycol) (PEG) canopy, exhibited fluidity with negligible vapor pressure and high thermal stability [44]. The addition of reactive acrylate-nanosilicas to a THPETA resin could result in softening rather than hardening upon forming nanocomposites [42]. For instance while the low loading of acrylate modified nanosilicas (1 wt%) increased the storage modulus (and decreased hardness), a high loading of acrylate modified nanosilica (50 wt%) decreased the storage modulus of the nanocomposite [42]. An ionic sulfonate SO${}_{2}$/im-PU matrix nanocomposite transitioned from a liquid-like to solid-like state as the loading of sulfonated SO${}_{2}$ increased. In particular, it exhibited a gel-like behavior for nanosilica loadings between 10 and 20 wt%. At low frequencies, the addition of nanosilica substantial increased the storage modulus and complex viscosity [58]. This behavior was directly related to the development of an percolated three-dimensional network of the silica nanoparticles within the im-PU matrix [58].

Moreover, in nanosilica composites with matrix imidazolium terminated chains (im-PLA) (im-P[CL-co-LA]), the storage modulus wass significantly influenced by the addition of im-PLA (Figure 13). In particular, the increase in im-PLA increased the storage modulus, in the lower frequency regime, by orders of magnitude and a gel-like plateau could be seen [59]. This behavior was attributed to the development of the ionic network of the im-PLA with the charged nanosilica [59]. The viscosity of ionic POSS hybrids was comparable to that reported for ionic liquids, and rapidly decreased as the temperature increased. The sorption of CO${}_{2}$ in POSS-based ionic fluids also reduced their viscosities [77].

In general, metals, such as gold and platinum, have a high melting point and cannot flow unless they are heated above 1000 °C. However, when metal nanoparticles, such as gold nanoparticles [121], are functionalized by grafting the carboxylate-terminated thiol and 11-mercaptoundecanoic acid (MUA), onto the surface of gold nanoparticles via chemisorption and are ionically tethered by PEG-substituted tertiary amine [84] can be inherited with improved fluidity and processability [75], and can, thus, be used as a lubricant, battery-cell plasticizer, etc. [84]. A range of different coronas (mercaptopropanesulfonate,1,3-diisopropylimidazolium, etc.) with different tertiary amines and quartery ammoniums (triisooctylamine,triisopentylamine, tripentylamine, etc.) as canopies have been applied to synthesize Au ionic nanofluids with diameters 6–20 nm [121].

The MoS${}_{2}$ nanofluids in the form of thin films can potentially be used for the lubrication of MEMs [87]. A Newtonian rheological behavior is observed due to the high graft density. This indicates that such nanofluids can have a stable lubricating performance and their rheological behavior could be tailored by changing the grafting density of the canopy and the type of inorganic cores or organic canopy [88]. The rheological behavior of magnetic Fe${}_{3}$O${}_{4}$ nanoscale ionic materials can easily be tuned [81]. It is found that long alkyl chains as surface modifiers can impart a lower viscosity and better flowability to the nanofluid [80]. The viscosity of both AMF and IMF nanofluids decreases with the increase in temperature because of the enhanced mobility of the canopy chains, while the descending rate of AMFs is slower than that of IMFs. This is mainly because more thermal energy is required to facilitate the disentanglement and molecule motion of the curved and entangled PEG chains in AMF nanofluids [81]. Dynamic moduli measurements as a function of shear strain reveal an essential difference between the two types of nanofluids. The storage modulus $G^{\prime }$, of both ionic nanofluids exhibits a linear viscoelastic regime at low shear strain and a yield strain of about 1%, however in the loss modulus $G^{\prime \prime }$ a maximum appears in IMFs which was absent in AMFs [81]. This is a distinct feature of soft glassy behavior. Such behavior can appear when the thickness of the organic shell is comparable to the dimension of the core [135].

#### 2.3.2. Anisotropic Nanofillers

**Figure 14:**
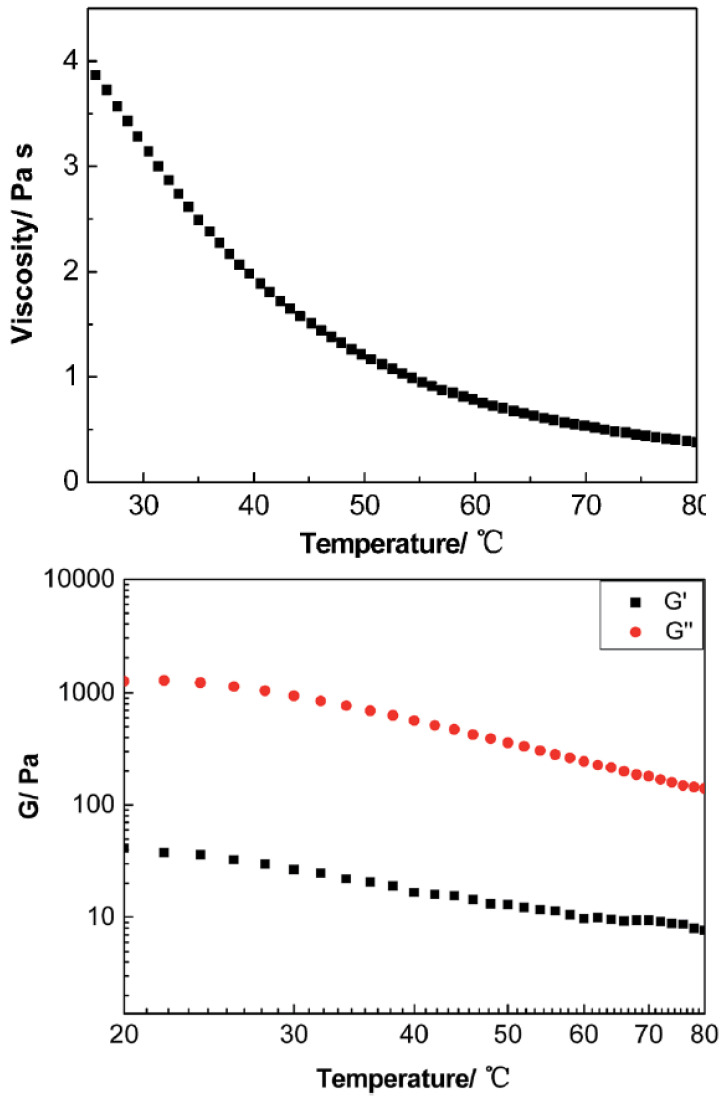
**Upper**: viscosity of the liquid-like MCWNT organic hybrid versus temperature at 10 $s^{-1}$. **Below**: the modulus of the liquid-like MWCNT organic hybrid versus temperature at 50$s^{-1}$. Reprinted with permission from Zheng et al. [97].

Interesting rheological behavior was also observed in ionic nanofluids containing anisotropic nanofillers. In particular, a MWCNT derivative synthesized by attaching Fe${}_{3}$O${}_{4}$ to the surface of MWCNT, employing tertiary amine terminated organosilanes as a corona, and sulfonated PEG salt as a canopy, displayed a liquid-like behavior at room temperature, where $G^{\prime \prime }$ was always higher than $G^{\prime }$ in the 20–80 °C temperature range, presenting a Newtonian rheological response at a low shear rate [97]. The viscosity of the derived MWCNT decreased dramatically as the temperature increased as is depicted in Figure 14.

In a study with graphene nanoparticle organic hybrid, a PEG sulfonated canopy was used. The concentration of graphene was 12.05 wt% [136]. Such graphene ionic material exhibited low viscosity at room temperature (67.6 Pa.s at 20 °C) [136]. In another study, sulfonation of GOs with sodium sulfanilic acid and ionic tethering with bulky amine-terminated Jeffamine (canopy) were prepared [108]. The supramolecular ionic liquid that was formed exhibited excellent solubility and amphiphilicity, confirming the liquid-like behavior by rheological measurements. If the canopy molecule was changed, such a nanoscale ionic liquid material could be a promising route for the fabrication of GO composites [108]. The loss modulus ($G^{\prime \prime }$) was higher than the storage modulus ($G^{\prime }$) [108] as was also observed in hydroxide perovskite (MnSn(OH)${}_{6}$) thread nanofluids [103]. The same rheological response for $G^{\prime \prime }$, $G^{\prime }$ with the viscosity gradually decreasing with temperature was observed for liquid-like nanocrystal-functionalized graphene oxide/Fe${}_{3}$O${}_{4}$ grafted with tertiary amine terminated organosilane, ionically tethered to sulfonated PEG salt [111]. Both moduli ($G^{\prime }$ and $G^{\prime \prime }$) of the GO derivate were approximately one order of magnitude larger than those of the neat Jeffamine. The shear thinning of the ionic GO derivative was noteworthy as it denoted its viscoelasticity. Both moduli ($G^{\prime }$ and $G^{\prime \prime }$) were independent of shear strain at low shear strain [108]. The nanoparticle concentrations had a dramatic effect on the rheological behavior [104]. The ionic G-OH nanomaterial with a solid content of 4.9 wt% showed almost Newtonian liquid behavior (such as the neat Jeffamine) with very little shear thinning. As the solid content of G-OH increased up to 11.6 wt% the $G^{\prime \prime }$ curve became parallel to $G^{\prime }$ curve, denoting the critical gel point. Both $G^{\prime \prime }$ and $G^{\prime }$ decreased with further increasing of the solid content and the ionic nanomaterial became solid-like [104]. Furthermore, calcium carbonate (CaCO${}_{3}$) ionic nanofluids showed fluidity (shear loss modulus $G^{\prime \prime }$ was higher than the storage modulus $G^{\prime }$) at room temperature, specifically from the soft organic shell that enabled the solid inorganic core to move smoothly [112].

### 2.4. Self-Healing

Beyond mechanical properties, in this section, we review the self-healing property of nanoscale organic hybrids and nanocomposites due to the dynamic ionic interactions located at the nanofiller and polymer matrix interface. Although this self-healing mechanism has been demonstrated in dynamic polymer networks [137], there are very few efforts that have proven the self-healing behavior in nanocomposites, through electrostatic interaction. According to Odent [58], the reversible nature of the ionic bonds [58] can simultaneously introduce a dynamic type of behavior to the material, providing opportunities for multi-responsive properties, such as high stiffness, toughness, self-healing, and shape-memory behavior. However, a nanocomposite with a low $T_{g}=-55$ °C has a high ductility at room temperature, and although it exhibits self-healing and shape-memory behavior, it has also a low stiffness (Young’s modulus) [58].

**Figure 15:**
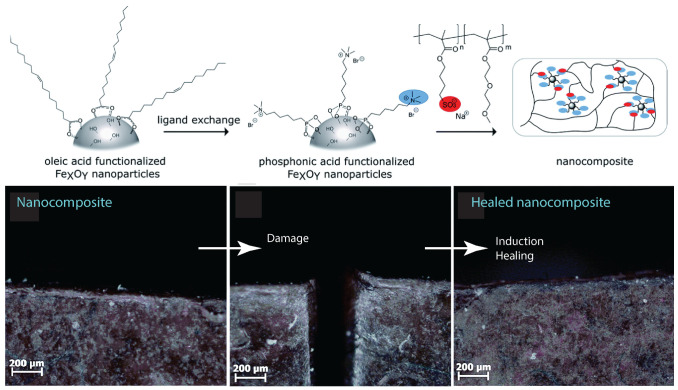
**Upper**: schematic of the synthesis process of the self-healing nanocomposite. **Below**: microscope image of the cut and healed nanocomposite samples. Reprinted with permission from Oberhausen and Kickelbick [123].

That nanocomposites exhibited a unique property profile that combined simultaneous improvements in stiffness, toughness, and extensibility. The presence of the dynamic and reversible imidazolium–sulfonate ionic interaction, resulted in the ability of the ionic nanocomposite to heal the scratches. Ionic nanocomposites containing 20 wt% of nanosilica-SO${}_{3}$H (sulfonated nanosilica) revealed a remarkable self-healing ability. After scratching the sample with a razor blade (scratch of ca. 500 µm depth and 160 µm width) and bringing the two cut pieces back into contact, the two faces spontaneously healed upon heating at 50 °C with a full scratch recovery after ca. 4 h [58]. Oberhausen and coworkers took advantage of the heating of iron oxide nanoparticles in an alternating magnetic field to induce the self-healing of ionic nanocomposites [123]. Overall, 8 nm superparamagnetic iron oxide nanoparticles were synthesized, by the thermal decomposition of iron (III) acetylacetonate, and used as the inorganic filler. The charge functionalization of the surface was introduced by ligand exchange with N,N,N-trimethyl-6-phosphonhexan-1-aminium bromide. The magnetic properties were mainly driven by their composition, size, morphology, and surface functionalization. Superparamagnetism was limited to magnetite particles at the nanoscale, particularly below 25 nm, thus potentially offering the opportunity for spatially resolved healing with an alternating magnetic field. The first step of the self-healing test consisted of exposing the damaged sample to a thermal treatment at 80 °C for 24 h leading to the complete healing of the scratch. Exposing the nanocomposites to an alternating magnetic field led to an increase in heating efficiency at the highest loading of iron oxide nanoparticles, i.e., 20 wt%. Induction heating was then used as a stimulus to initiate the healing process by adjusting the field strengths to reach a macroscopic temperature of 55 °C. According to microscope observations, a complete healing was observed after pressing the edges together, after 24 h and 48 h of healing time (Figure 15). The impact of magnetic nanoparticles on the healing process was confirmed by applying an alternating magnetic field to the polymer matrix where no healing was observed. [123]

Solvent-free ionic molybdenum disulfide (MoS${}_{2}$) nanofluids showed self-healing lubricating behaviors [87]. Homogeneous and stable solvent-free ionic MoS${}_{2}$ nanofluids were obtained by the surface functionalization and ionically tethering of nanoscale graphite-like MoS${}_{2}$ from hydrothermal synthesis. The self-healing property was assessed by in situ images of scratches taken by SPMe which was equipped with a nanomechanical tester. The scratches under different normal loads were applied on the surface of the ionic nanocomposites. The comparison of the two images with the same scratch under 400 mN, captured at 200 s intervals, revealed the self-healing property of the nanofluids after 200 s. These findings fully support that the MoS${}_{2}$ nanofluids, in the form of thin films, can protect the substrates from scratching and wear.

**Figure 16:**
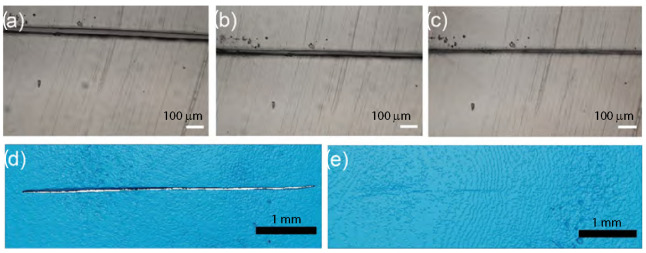
Self-healing behavior of ionic PDMS—nanosilica composites, (**a**) before and after thermal treatment (80 °C) in a humid environment for (**b**) 30 min and (**c**) 1 h. Micro-computed X-ray tomography (micro-CT) results of the same specimen confirm this behavior, showing the entire scratch (**d**) before healing and (**e**) after healing for 1 h at 80 °C in a humid environment. Reprinted with permission from Mugemana et al. [120].

Cross-linking PDMS through ionic interactions promised the possibility of self-healing behavior given their reversible nature [120] as depicted in Figure 16. The self-healing ability was tested by creating a scratch on the surface of various ionic PDMS nanocomposite films and monitoring the scratch over time, both by optical microscopy and micro-computed X-ray tomography (micro-CT). A healing temperature of 80 °C was chosen based on the observation of a glass transition between 70 and 85 °C by the ionic groups in the system. It was suggested that heating the ionic nanocomposite films to temperatures close to the glass transition would induce scratch healing, as previously demonstrated in ionic polyurethane-based nanocomposites [58]. The ionic PDMS nanocomposite containing low molecular weight PDMS (6.5 kg/mol) with a loading of 10 wt% nanosilica possessed the ability to heal rapidly in a humid environment, apparently due to the higher mobility of nanosilica in a non-entangled PDMS matrix. A decrease in scratch width in this system was observed after 30 min (Figure 16b), and after one hour, the complete healing of the scratch was observed (Figure 16c). This was confirmed with micro-CT analysis, which revealed a scratch 5 mm and 25 ± 5 µm wide prior to healing (Figure 16d) vs. the near-complete disappearance of the scratch following healing in a warm and humid environment (Figure 16e). The recovery of mechanical properties after damage and healing was assessed by a mechanical testing analysis showing a recovery of 73 ± 20%. These results confirmed the sensitivity of the electrostatic (ionic) interactions of those systems to the presence of moisture, and their very strong impact on nanoparticle mobility and polymer chains relaxation.

## 3. Simulations

Although in the field of polymer–nanoparticle mixtures, molecular simulation has been implemented extensively to study structure, dynamics, and properties, this is not the case for nanoscale ionic organic hybrids and ionic nanocomposites. In particular, there is no atomistic molecular simulation study that explores the behavior of such nanomaterials. Neither has there been any simulation effort of anisotropic ionic nanofluids or nanocomposites. Nevertheless, the molecular simulation methods (both atomistic and coarse-grained) are appropriate techniques to study the liquid-like behavior of such nanomaterials, and explore the structure and dynamics of canopies (or ionic polymers) near the nanoparticle surface or functionalized nanoparticles.

### 3.1. Structure and Dynamics

**Figure 17:**
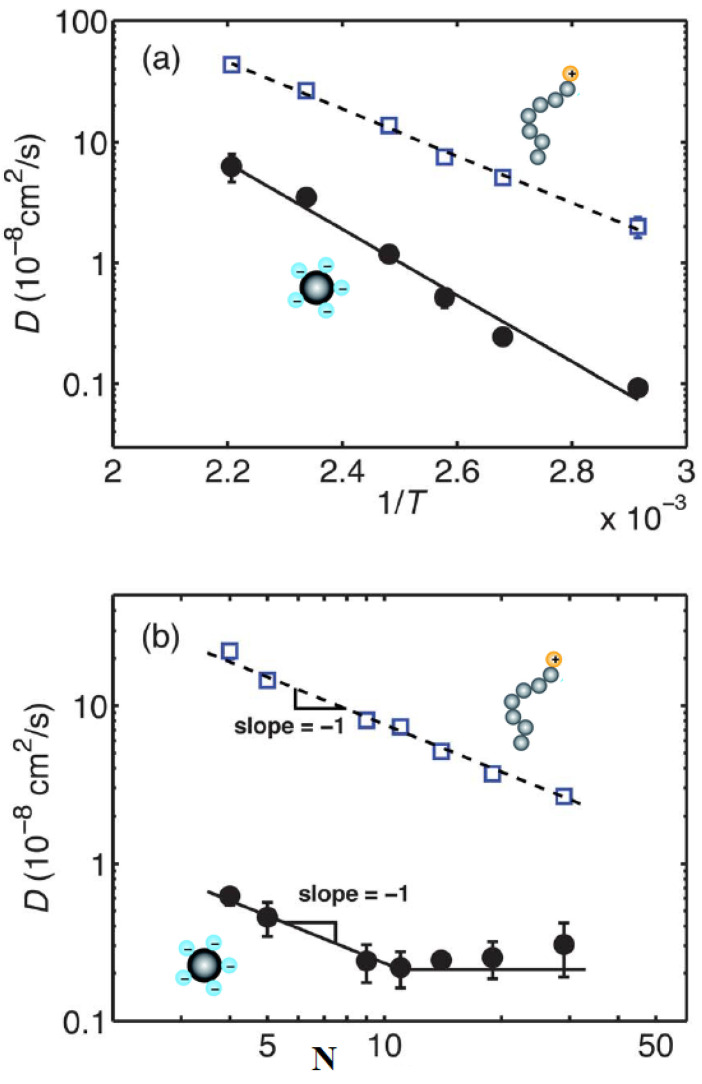
Self-diffusion coefficients of the cores (solid circles •), and chains (open squares □) as functions of (**a**) inverse temperature for chain length N=14, and surface beads on the core g=8; (**b**) chain length *N* at T= 100 °C with g=8. Solid and dashed lines in (**a**) are Arrhenius expressions fitted to the cores and the chains, respectively. The dashed line in (**b**) is fitted to the Rouse model and the solid line to the theory of Ref. [145]. Reprinted and enhanced by thumbnails with permission from Hong and Panagiotopoulos [143].

There have been initial efforts to simulate the structure and dynamics of nanoparticle organic ionic liquids [138,139,140]. For instance, initial theoretical efforts, using the density functional theory of point particles or finite hard cores with bead-spring-attached oligomeric chains, of solvent-free nanoparticle organic hybrid materials were used to derive the radial distribution function and structure factor of nanoparticles [138]. For different nanoparticle core volume fractions and radii of gyration of oligomers, the static structure factor goes to zero for zero wavenumber, meaning that the core nanoparticle and its oligomers fill a volume of space that sterically excludes exactly one neighboring nanoparticle [138]. Moreover, it is hypothesized that an “onion”-like model for nanosilica ionic materials, where the polymeric cations are kept around the nanoparticle anion due to the long range of electrostatic interactions relatively long-range coulomb interactions. The Debye length, (1/κ= 1–10 nm) for the “onion”-like model, scales linearly with the square root of the dielectric constant ($\epsilon_{r}$) and temperature and inversely with the square root of the ionic strength. This implies that for a polymer ($\epsilon_{r}=$ 2–10) and assuming the “onion”-like conformation the ionic concentration should be $10^{-2}-10^{-5}$ ions/nm${}^{3}$, which was confirmed by PFG-NMR experiments [61]. While the self-diffusion coefficient, for long periods, of the cores and steady low-shear viscosity of the system obtained from the analysis are similar to hard sphere suspensions at a higher core volume fraction or with longer oligomeric chains [141]. The tethered molecules incur an entropic penalty to fill the space at low-volume fractions and with shorter chains [141]. Furthermore, using an improved coarse grained model, the structure and transport properties (diffusivity, viscosity, conductivity) of solvent-free ionically grafted nanoparticles were predicted using microsecond time simulations [142,143]. Although the electrostatic interactions between oppositely charged ions at contact are greater than the thermal energy, $k_{B}T$, the chain dynamics at intermediate time scales are dominated by chain hopping between core nanoparticles. Moreover, neutral core nanoparticles with tethered chains diffuse faster than the ionic core nanoparticles [142]. The dependence of the transport properties on temperature follows an Arrhenius type of relation [143] as depicted in Figure 17a. In particular, the diffusion coefficient of the cores reaches a plateau beyond a certain length of the canopy (Figure 17b). More coarse-grained simulation efforts on nanoscale ionic materials investigate the structure and dynamics of an ionically tethered canopy. In a system without added salt, the charged terminal groups of the canopies adsorb strongly on oppositely charged walls, due to the attractive electrostatic interaction. Such ionic canopies are stretched and do not desorb from the oppositely charged surface [144]. In a system that contains electrolyte ions, the counterions adsorb on the charged walls, thus causing some ionic canopies to desorb from the charged surface. The desorbed polymers adopt conformations similar to those in bulk. There is an interplay of electrostatic and entropic interactions that determine the structure and dynamics of the canopies [144].

**Figure 18:**
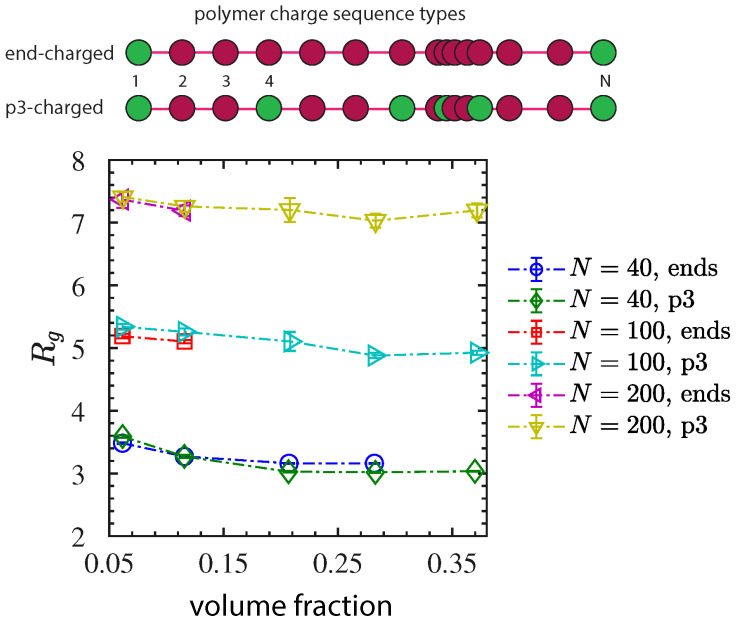
Radii of gyration $R_{g}$ (in units of bond length) of ionic polymers versus nanoparticle volume fraction for dispersed systems, by equilibrium molecular dynamics. Reprinted with permission from Moghimikheirabadi et al. [146].

However, these initial efforts were based on ionically grafted nanoparticles ionically bonded to oligomers and did not contain an entangled polymer matrix. In the most recent coarse-grained molecular dynamics (MD) simulations of an ionic polymer nanocomposite model, the authors found that nanoparticle dispersion can indeed be achieved due to the insertion of an electrostatic charge [129,146]. Moreover, nanoparticle diffusion [147,148] in the polymer matrix slowed down due to this electrostatic charge, and the ionic nanoparticles moved according to a hopping mechanism [129]. Based on their experimental findings, Odent et al. [58,60] suggested that ionically functionalized nanosilicas are mobile inside of a PU matrix and simultaneously form ionic crosslinks between the PU polymers, creating local regions of enhanced strength and enhanced energy dissipation. To test the hypothesis, the role of ionic interactions and nanoparticle, which mimic nanosilica, loading [8] on structure and dynamics for end-charged and regularly charged polymers have been studied via coarse-grained simulations. These simulations revealed that the ionic crosslinks between polymers and nanoparticles were drastically influenced by the nanoparticle loading [146]. The ionic polymer conformations [149], as described by their radii of gyration, were altered by the loading of charged nanoparticles, while the radius of gyration of charged entangled polymers were unperturbed by the presence of charged nanoparticles [146], as can be seen in Figure 18.

**Figure 19:**
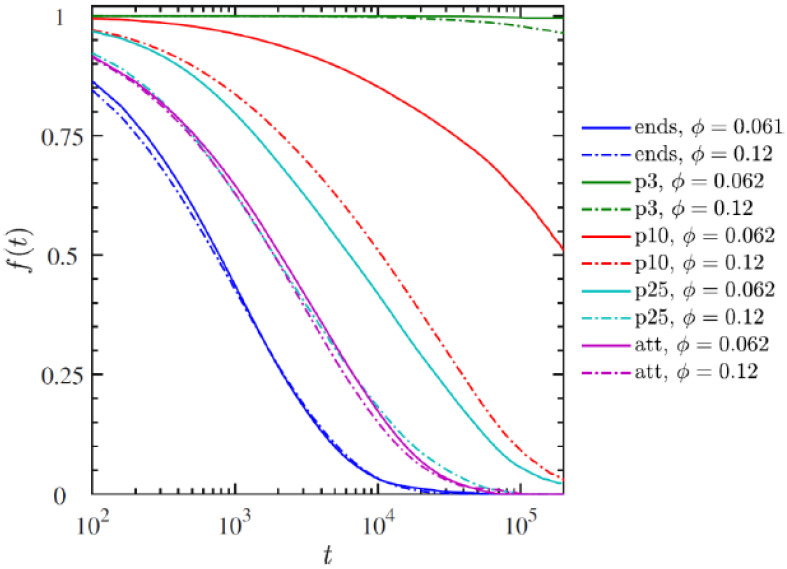
Survival probability f(t) versus reduced time *t* of ionic crosslinks for different nanoparticle volume fractions, polymer charge density, and localization of charges. Reprinted with permission from Mugemana et al. [120].

Both the dynamic behavior of ionic nanoparticles and polymers and the amount of temporary ionic crosslinks (Figure 19), were found to depend on the electrostatic strength (ratio of Bjerrum length and characteristic distance between charged monomers) [120]. Simulations showed that the life time of ionic crosslinks is in the order of ns [120], which agreed with experimental observations by Jespersen et al. [35,61,62]. Polymer–polymer entanglements started to be reduced beyond a certain nanoparticle volume fraction. The dynamics of ionic nanoparticles and polymers was very different to their neutral counterparts [146]. Specifically, ionic nanoparticle dynamics was being enhanced in entangled polymer matrices and accelerated with the nanoparticle loading.

### 3.2. Mechanical Properties

**Figure 20:**
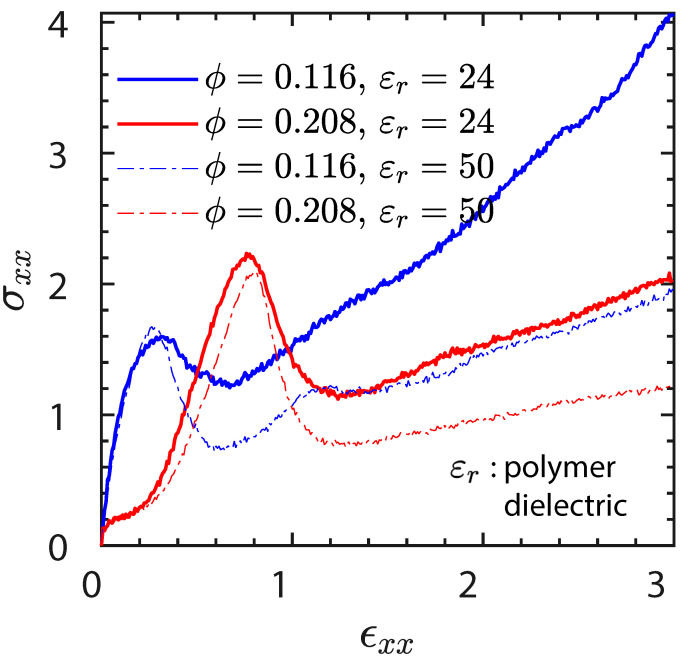
Stress–strain behavior of ionic modelled nanocomposites for different nanoparticle volume fractions and dielectric polymer medium constants, by NEMD. The stress component $\sigma_{xx}$ is reported in Lennard–Jones units. Reprinted with permission from Moghimikheirabadi et al. [150].

Coarse-grained nonequilibrium molecular dynamics (NEMD) simulations [150] investigated the mechanical response of ionic nanocomposites for polymers carrying charges on every third monomer and of different dielectric constant, that influenced the electrostatic strength (via Bjerrum length). An increase in stiffness and toughness of the ionic nanocomposites was observed upon increasing the engineering extensional strain rate, as can be seen in Figure 20, and in agreement with experimental observations [58]. The excellent toughness of the ionic nanocomposites was shown to originate from the electrostatic interaction between ionic polymers and nanoparticles, and was not due to the nanoparticles mobility, as was speculated in Ref. [58] or the presence of polymer–polymer entanglements [150].

These studies moreover provide a microscopic insight into the structural and conformational changes accompanying the mechanical properties. In particular, systems exhibit an overshoot in the number of entanglements at moderate strains prior to entanglement loss at large strains. The variation of mean pore radius (which is the largest sphere that can be inserted into the matrix without any overlap with existing nanoparticles [150]) is resolved as function of strain and extension rate, and the stress maxima are shown to be correlated with a disorder parameter that quantifies the degree of crystalline order of the embedded ionic nanoparticles.

## 4. Conclusions

Overall, the choice of corona, canopy, and cores can determine the properties of an ionic nanoparticle organic hybrid and this can, therefore, add tailorability and functionality to such ionic nanomaterials. Using polymeric chains with specific functional groups can enhance diffusivity and selectivity towards certain gas molecules, thus promoting gas separation. For instance, the canopy structures and their steric interactions with CO${}_{2}$[49] and sulfur dioxide (SO${}_{2}$) that exist in flue gas can interact with the ether groups, owing to its Lewis acidic nature, of the polymeric canopy and, thus, can change the CO${}_{2}$ packing behaviors in nanoscale organic hybrids [151]. In order to achieve a more effective CO${}_{2}$ capture material, different functional groups that have a strong chemical affinity with CO${}_{2}$ can be added to the polymeric canopy (enthalpic contribution), and various steric interactions can be induced by attractive/repulsive interactions between the nanocores and canopies (entropic contribution) [63]. In addition, the enhanced chemical and thermal stability of such tethered polymers in these hybrids could provide a great potential for reactive and separation systems, including flow batteries [110] and CO${}_{2}$ capture [64]. In ionic MXene nanomaterials, using a different alkaline oligomer can advance their development, leading to applications using catalysis and photothermal conversion [117]. Nevertheless, the electrostatic attraction between nanoparticles and polymers has become very efficient in improving the nanoparticle dispersion state which is a prerequisite of improved material properties. In particular, the viscosity of the ionic MWCNT derivative is dramatically reduced with temperature denoting a possible future application in the fabrication of ionic MWCNT/polymer nanocomposites with improved processability.

Furthermore, the choice of canopy or functionalized polymer matrix that can interact ionically with the oppositely charged nanoparticle is critical in order for it to lead to derivatives with tunable properties or to achieve the desired mechanical, rheological, electrical [83], self-healing, and shape-memory properties. In particular, different parameters of the polymeric matrix can influence these properties. The $T_{g}$ of the polymer, polymer molecular weight, polymer dielectric constant, localization of the charges on the polymer chain and polymer charge density can drastically influence polymer dynamics, thus assimilating the ionic nanomaterial (nanocomposite) with the desired properties. Atomistic MD simulations will be necessary to identify the conformation and dynamics of ionic canopy or polymer matrix in the interphase and how these are influenced by the nanoparticle surface or temperature. Further NEMD studies could shed light on the linear viscoelastic, shear behavior, and reinforcement mechanisms of ionic nanocomposites.

## Data Availability

Not applicable.
